# Association between ustekinumab therapy and changes in specific anti-microbial response, serum biomarkers, and microbiota composition in patients with IBD: A pilot study

**DOI:** 10.1371/journal.pone.0277576

**Published:** 2022-12-30

**Authors:** Filip Rob, Dagmar Schierova, Zuzana Stehlikova, Jakub Kreisinger, Radka Roubalova, Stepan Coufal, Martin Mihula, Zuzana Jackova, Miloslav Kverka, Tomas Thon, Klara Kostovcikova, Lukas Bajer, Pavel Drastich, Jana Tresnak Hercogova, Michaela Novakova, Martin Kolar, Martin Vasatko, Milan Lukas, Helena Tlaskalova-Hogenova, Zuzana Jiraskova Zakostelska

**Affiliations:** 1 Second Faculty of Medicine, Dermatovenerology Department, University Hospital Bulovka, Charles University, Prague, Czech Republic; 2 Laboratory of Cellular and Molecular Immunology, Institute of Microbiology of the Czech Academy of Sciences, Prague, Czech Republic; 3 Faculty of Science, Department of Zoology, Charles University, Prague, Czech Republic; 4 Hepatogastroenterology Department, Institute for Clinical and Experimental Medicine, Prague, Czech Republic; 5 ISCARE a.s., IBD Clinical and Research Centre, Prague, Czech Republic; 6 General University Hospital and First Faculty of Medicine, Institute of Medical Biochemistry and Laboratory Diagnostics, Charles University, Prague, Czech Republic; University of Minnesota Twin Cities, UNITED STATES

## Abstract

**Background:**

Ustekinumab, is a new therapy for patients with IBD, especially for patients suffering from Crohn’s disease (CD) who did not respond to anti-TNF treatment. To shed light on the longitudinal effect of ustekinumab on the immune system, we investigated the effect on skin and gut microbiota composition, specific immune response to commensals, and various serum biomarkers.

**Methodology/Principal findings:**

We recruited 11 patients with IBD who were monitored over 40 weeks of ustekinumab therapy and 39 healthy controls (HC). We found differences in the concentrations of serum levels of osteoprotegerin, TGF-β1, IL-33, and serum IgM antibodies against *Lactobacillus plantarum* between patients with IBD and HC. The levels of these biomarkers did not change in response to ustekinumab treatment or with disease improvement during the 40 weeks of observation. Additionally, we identified differences in stool abundance of uncultured *Subdoligranulum*, *Faecalibacterium*, and *Bacteroides* between patients with IBD and HC.

**Conclusion/Significance:**

In this preliminary study, we provide a unique overview of the longitudinal monitoring of fecal and skin microbial profiles as well as various serum biomarkers and humoral and cellular response to gut commensals in a small cohort of patients with IBD on ustekinumab therapy.

## Introduction

Inflammatory bowel diseases (IBD), including ulcerative colitis (UC) and Crohn’s disease (CD), are severe chronic relapsing disorders of the gastrointestinal tract, whose etiology and pathology remain largely unknown despite intensive research [1]. Rather than being a single disease, or two diseases (CD and UC), IBD is increasingly understood as a multifactorial disorder that is initiated and exacerbated by influences such as genetic susceptibility, environmental factors, aberrant immune response, and alteration in the intestinal microbiota [2].

The worldwide incidence of IBD is increasing, especially among young adults and children in industrialized countries. Biological therapy targeting the immune system components is successfully used in IBD treatment. The most commonly used biologics are tumor necrosis factor alpha (TNF-α) inhibitors, which demonstrate high efficacy and long-term improvement in the quality of life in patients with IBD (2). However, one-third of patients receiving anti-TNF inhibitors remain unresponsive [3,4].

Ustekinumab is a fully humanized IgG1κ monoclonal antibody that binds to the common p40 subunit of interleukin 12 (IL-12) and interleukin 23 (IL-23). This prevents IL-12 and IL-23 cytokines from binding to their receptors, thereby reducing the maturation and proliferation of Th-1 and Th-17 cells. Recently, ustekinumab has become a common choice for IBD treatment, especially during refractory disease course in patients with CD [5]. Recent data suggest that treatment with ustekinumab has a low risk of adverse events in short-term maintenance [6]. In contrast to anti-TNF inhibitors, ustekinumab treatment results in a much lower rate of adverse skin reactions and can even resolve them after switching from another drug [7,8]. In addition, ustekinumab treatment, especially in moderately to severely active UC, is more likely to achieve better clinical response, clinical remission, and endoscopic mucosal healing, compared to anti-TNF inhibitors [9]. Ustekinumab has also been reported to be effective in patients with CD who did not respond to anti-TNF treatment [10]. There is currently an intensive effort to predict the primary response to ustekinumab therapy based on age, gender, weight, surgery, previous anti-TNF therapy, or disease-related factors (duration, location). However, these studies have produced equivocal results [11,12].

The intestinal inflammation in IBD is accompanied by a breach of the intestinal barrier and dysbiotic microbiota. Various studies have reported reduced microbial diversity in patients with active IBD [13] and a correlation of specific bacterial composition with disease severity [14,15]. The exposure of the mucosa to bacterial antigens could lead to stimulation of specific immune response. Therefore, serum antibodies against specific bacteria could serve as predictive markers of gut barrier function and gut microbiota composition, which could be modulated during treatment. Although homeostatic levels of bacteria-specific IgG are present to protect the host from systemic infection by commensal bacteria [16], several studies have reported an increase in non-specific and specific gut bacteria IgG levels in serum of patients with IBD [17–21]. In addition, several studies have suggested a role of gut fungi in the pathogenesis of IBD [22], with the abundance of *Candida* consistently elevated in patients with CD [23,24]. T cell response against the resident gut commensals is present under physiological conditions, but the cytokine repertoire produced is altered during inflammation. Therefore, we measured levels of specific cytokines upon stimulation with common gut commensals [25]. Latest studies suggest that patients with IBD (our unpublished data) and psoriasis [26] differ from healthy controls in their skin microbiome composition and diversity suggesting a communication along the gut-skin axis. Therefore, we included skin microbiome analysis to broaden the previous findings in a specific group of patients.

This pilot study aimed to describe the longitudinal changes in potential biomarkers that could predict and monitor the therapeutic efficacy of ustekinumab treatment in patients with IBD. We evaluated the clinical status of patients with IBD and analyzed various molecules from the serum as well as gut and skin bacterial and fungal composition. We correlated the microbiome data and serum biomarkers with the clinical outcome of patients after 40 weeks of treatment with ustekinumab.

## Materials and methods

### Patients and sample collection

Patients included in the study were recruited at the IBD Clinical and Research Centre, Prague, Czech Republic from November 2018 to December 2020. All participants in our study were Caucasians. The patients were diagnosed with IBD and assigned ustekinumab treatment according to the guidelines of the European Crohn’s and Colitis Organization (ECCO) [27]. In total, 57 stool samples, 44 skin samples, and 40 serum samples from multiple time points during the ustekinumab treatment were collected from 11 patients with IBD (S1 Table). During each visit (week 0, 8, 16, 24, 32, and 40), medical history, disease severity, responsiveness to treatment, and the following clinical parameters were monitored: Harvey-Bradshaw index (HBI) in patients with CD and partial Mayo score (pMayo) in patients with UC; C-reactive protein (CRP); fecal calprotectin (FC); ustekinumab serum trough levels (TLs); anti-ustekinumab serum antibodies (anti-UST); indication for biological treatment; past biological therapy. HBI or pMayo were calculated by specialized IBD nurses during patients’ visits at the outpatient department. HBI and pMayo with values ≤4 and ≤1, respectively, were considered disease remission. Healthy control subjects were recruited at the Institute of Clinical and Experimental Medicine (IKEM). The exclusion criteria for healthy subjects were a gastrointestinal or dermatologic diagnosis and the use of antibiotics within 3 months prior to the sampling. In total, 43 stool samples, 33 skin samples, and 38 serum samples were collected from 39 healthy control individuals (S1 Table). Written informed consent form was obtained from all study participants. This study was approved by the Ethics Committee of ISCARE (Nr2015/Ia) and IKEM (Nr2015/Ia). All samples were stored at -80°C until processing.

### Stool sample collection and DNA extraction

Stool samples were freshly collected by the participants into standardized sterile collection tubes prior to the clinic visit. All samples were delivered within 6h after collection, immediately frozen at -80°C and further processed as described previously [28]. Patients provided the stool samples at the beginning of ustekinumab therapy (baseline = week 0) and at weeks 8, 16, 24, 32, and 40.

### Skin sample collection and DNA extraction

Skin swab samples were collected at the University Hospital Bulovka (Prague, CZ) from patients enrolled in ISCARE as mentioned above. The swab samples were taken from the retroauricular crease by an accredited dermatologist using the protocol established at the Human Microbiome Project Consortium to minimize collection bias [29]. Briefly, swab samples were taken from a 2 × 2 cm area using flocked swabs (FLOQSwabsTM COPAN Diagnostics Inc., United States) soaked in sterile SCF-1 buffer [50 mM Tris buffer (pH 7.6), 1 mM EDTA (pH 8.0), 0.5% Tween 20]. All collected swabs were immediately frozen at −80°C. Extraction of total DNA from swabs and scrapings was performed using DNeasy PowerBiofilm Kit (Qiagen, Hilden, Germany) with minor changes in the protocol exactly as described previously [26]. Patients provided the skin swab samples at the beginning of ustekinumab therapy (baseline = week 0) and at weeks 8, 16, 24, 32, and 40.

### Preparation of bacterial lysates

Selected bacteria were obtained from DSMZ (Deutsche Sammlung von Mikroorganismen und Zellkulturen GmbH, Braunschweig, Germany) and cultured as recommended. Specifically, *Lactobacillus plantarum* CCDM 182 (ATCC Medium No. 416); *Bifidobacterium adolescentis* CCUG 18363 (DSMZ medium No. 58.); *Blautia coccoides (*ATCC Medium No. 1490); *Roseburia intestinalis* L1-82 (ATCC Medium No. 2695); *Eubacterium rectale* ATCC 3365 (ATCC Medium No 1703); *Faecalibacterium prausnitzii* A2-165 (DSMZ medium No. 1611); *Ruminococcus flavefaciens* (ATCC Medium No. 1365 E); *Bacteroides thetaiotaomicron* VPI 5482 (ATCC Medium No. 1490); *Prevotella ruminicola* M384 (ATCC Medium No 1703); *Escherichia coli* K6 (Merck, Kenilworth, NJ, USA, L3022). All bacteria except *E*. *coli* K6 were grown under anaerobic conditions. Ten bacterial lysates were prepared as described previously and then used for peripheral blood mononuclear cells (PBMCs)stimulation or for antigen coating in an indirect ELISA (see details below) [30,31].

### Stimulation of peripheral blood mononuclear cells with commensal antigens

PBMCs were isolated, cultivated with 10 lysates from 10 different commensal bacteria and stained for subsequent analysis by flow cytometry as previously described [32].

### Antibody and biomarker levels detection by ELISA

Based on our previously published research of patients with IBD [33], we selected specific markers listed in the Supplementary Materials (S2 Table). All ELISA assays were performed according to the manufacturer’s recommendations.

Serum concentrations of total immunoglobulin G (IgG), immunoglobulin A (IgA), and immunoglobulin M (IgM) were determined using commercial kits according to the manufacturer’s instructions (Invitrogen, Waltham, MA, USA). The serum concentrations of anti-bacterial IgG, IgA, and IgM isotypes were analyzed by an in-house developed indirect ELISA exactly as described previously [32]. All patient serum samples were diluted at 1:200. Antibody levels were reported in arbitrary units (AU), where a selected reference serum sample was applied on each ELISA plate and its mean value of OD (450–650 nm) was used as thousand arbitrary units (1000AU).

### Amplicon sequencing of skin and stool samples

The V3V4 region of the 16S rRNA gene and the fungal ITS1 region covered by specific degenerate primers with barcodes (341F GTCCTACGGGNGGCWGCAG and 806R GGACTACHVGGGTWTCTAAT) and (F GTAAAAGTCGTAACAAGGTTTC and R AAGTTCAAAGAYTCGATGATTCAC), respectively, were chosen as representative sequences for taxonomic identification. The amplification of stool DNA was performed with the KAPA HiFi HotStart Ready Mix (Roche, Penzberg, Germany) as follows: initial denaturation step 3 min at 95°C followed by 25 cycles at 95°C for 30 s, 55°C for 30s, 72°C for 30 s with a final elongation step at 72°C for 5 min using 12.5 ng DNA. The amplification of skin DNA was performed with HiFi polymerase (Roche, Penzberg, Germany) as follows: initial denaturation step 3 min at 94°C followed by 33 cycles at 94°C for 1 min, 55°C for 5 s, 72°C for 2 min with a final elongation step at 72°C for 10 min using 5μl DNA. PCR products were checked using QIAxcel Advanced capillary electrophoresis (QIAgen, Hilden, Germany). Triplicates of the amplicons were pooled and normalized with the SequalPrep™ Normalization Plate Kit (ThermoFisher Scientific, Waltham, MA, USA), concentrated (Eppendorf centrifugal vacuum concentrator), and purified with the DNA Clean & Concentrator Kit (Zymo Research, Irvine, CA, USA). Subsequently, the amplicon libraries were ligated with sequencing adapters using the KAPA HyperPlus Kit (Roche, Penzberg, Germany), pooled in equimolar concentrations, and sequenced using the MiSeq Reagent Kit v3 (2 x 300 bp) at the CEITEC Genomics Core Facility (Brno, Czech Republic).

### Bioinformatics analysis

Reads were quality filtered with Cutadapt (version 1.15), joined with Fastq-join (version 1.3) and demultiplexed using a custom R script. Sequences were trimmed at 440bp, denoised and amplicon sequence variants (ASVs) were generated with a single command using Qiime2 DADA2 plugin (version 2021.2) with default parameters. Qiime2 (version 2021.2) pipeline was used to calculate alpha and beta diversities [34]. Bacterial taxonomy was assigned with the vsearch classifier against the Silva database (release 138) with 99% similarity. Only the forward reads of ITS sequences were used and fungal taxonomy was assigned using blast against the Unite database (version 8) with 97% similarity. The sequencing data are available at the Sequence read archive under accession number PRJNA757573.

### Statistics

Biomarkers: Principal Component Analysis (PCA) and Multivariate Distance Matrix Regression (MDMR) [34] were used to assess overall differences in biomarker profiles between healthy controls and patients with IBD as well as within the cohort of patients with IBD sampled during the 40 weeks after the initiation of the biological treatment. Biomarker concentrations were square root-transformed prior to analyses to normalize their distribution. For MDMR, we used a matrix of Euclidean distances among samples calculated based on concentrations of all biomarkers as the response. MDMR models were controlled for pseudoreplications due to repeated sampling of the same individuals through random effects.

Using linear mixed effect models (LMM) with individual identity modeled via random intercepts, we explored each biomarker’s variation between patients with IBD and HC as well as longitudinal changes of biomarker concentrations in patients with IBD during the course of biological treatment. For each biomarker, we fitted (1) the null model (i.e., fitting just the intercept) and models that considered the effect of the week as (2) a linear continuous predictor, (3) a quadratic polynomial term, (4) a piecewise polynomial term fitted via B-splines (R package splines) or (5) as a categorical predictor. Performance of these alternative models was compared using the Akaike Information Criterion (AIC) and the significance of temporal changes was assessed based on the deviance change between the null model and the best performing non-null model. Biomarker concentrations were Box-Cox transformed prior to analysis to achieve normal distribution of the residuals. LMMs were also used to test for differences in biomarker concentrations between patients with IBD and healthy controls. The Q-value method [35] was used to adjust p-values for multiple testing, if necessary.

Microbiota: We studied variation in microbial alpha diversity among healthy controls and patients with IBD sampled during the 40 weeks of biological treatment using LMM with Gaussian error distribution and individual identity included as a random factor. All alpha diversity indices, except for the Shannon entropy, were log10 transformed prior to analysis, to achieve normal distribution of the residuals.

Principal Coordinate Analyses (PCoA) were used to explore the variation in microbiota composition among samples. For analyses of bacteriome profiles, PCoA was conducted for Jaccard, Bray-Curtis, and both weighted and unweighted UniFrac dissimilarities, while only the non-phylogenetic Jaccard and Bray-Curtis distances were used for mycobiome analyses.

Next, MDMR was applied to test for systematic changes in microbiota composition of patients with IBD during the 40 weeks of treatment. Additional MDMR was also fitted to assess the differences in microbiota composition between patients with IBD and healthy controls. To account for the fact that patients with IBD were sampled repeatedly, individual identity was modeled by random effects.

To test for systematic differences in interindividual variation between patients with IBD and healthy controls and patients at different treatment stages, we calculated the distance of each sample from the centroid of the respective group using the betadisper function in the R package vegan and used it as the response in a LMM with individual identity included as a random factor.

Differentially abundant taxa were identified by ANCOM v2.1, a modified version of ANCOM [36], that allows pairwise comparisons and adjustment for covariates. To control for repeated measures in time, the individual was considered as a random effect.

## Results

### Patient cohort characteristics

Our study cohort comprised 11 patients with IBD who underwent biological therapy with ustekinumab and 39 HC subjects. In total, 57 stool samples, 44 skin samples, and 40 serum samples were collected from the study subjects before therapy induction at week 0 and at weeks 8, 24, 32, and 40 during the treatment (S1 Table). Patients received the first dose (260, 390, or 520 mg depending on their weight) intravenously and then continued with a maintenance dose of 90 mg according to the manufacturer’s protocol. Anthropometric and clinical characteristics of our study cohort, including HC subjects and patients with IBD, at the study baseline and endpoint are summarized in Table 1. For extended additional information about the characteristic of our study cohort see S3 Table. Basic clinical parameters, such as CRP, WBC, PLT, ferritin, Hb, and FC were measured (Table 1). The elevated levels in patients with IBD compared to HC point to ongoing inflammation. At baseline (week 0), 50% of patients with IBD were in clinical remission. The ECCO guidelines for ustekinumab prescription includes insufficient response to the conventional treatment, for this reason, the patients in our cohort were not drug naïve (S3 Table). None of the patients experienced an adverse skin reaction or had anti-ustekinumab antibodies.

**Table 1:** Summary of anthropometric and clinical parameters from patients with IBD and healthy controls at the study baseline (week 0) and endpoint (week 40). In total, we collected samples from 11 patients with IBD (10 samples at baseline, 8 samples at endpoint) and 39 healthy controls. Comparisons between patients with IBD and HCs were evaluated by the Mann-Whitney U test. Changes during ustekinumab treatment (week 0 vs. week 40) were evaluated by Wilcoxon matched-pairs signed rank test. Bonferroni method was used to correct for multiple hypothesis testing. Medians are reported with the first and third quartiles in parentheses. n (number of participants), CD (Crohn’s disease), UC (ulcerative colitis), HC (healthy control), HBI (Harvey-Bradshaw Index), pMayo (partial Mayo score), CRP (C-reactive protein), FC (fecal calprotectin), Hb (hemoglobin), PLT (platelet count), WBC (white blood cells), ns (not significant), NA (not available).

	HC (*n* = 39) week 0	IBD (*n* = 10)	IBD (*n* = 8)	Δ IBD	IBD vs HC
		week 0	week 40	(*p*-val)	(*p*-val)
**Male: female**	14:25	6:4	4:4	-	-
**Age**	37.0 (27.5, 43.0)	40.0 (33.5, 52.0)	40.0 (35.8, 49.5)	-	-
**BMI**	22.9 (20.9, 27.0)	25.1 (20.8, 27.2)	25.5 (23.8, 26.3)	-	-
**Disease duration in years**	-	10.5 (6.3, 13.5)	10.5 (9.5, 14.3)	-	-
**Age at diagnosis**	-	31.0 (25.5, 41.5)	31.0 (26.5, 37.5)	-	-
**Diagnosis CD: UC**	-	8:2	6:2	-	-
**Patients in remission**	-	5/10	5/8	-	-
**HBI/pMayo**	-	4.0 (2.5, 5.5) / 6.5 (5.3, 7.8)	3.0 (1.5, 4.5) / 1.5 (1.3, 1.8)	ns / ns	-
**CRP**	1.3 (0.4, 2.1)	7.3 (4.3, 9.9)	2.7 (2.2, 4.1)	ns	< 0.001
**WBC**	5.7 (4.9, 6.6)	8.2 (6.0, 9.8)	7.3 (6.9, 9.5)	ns	< 0.001
**PLT**	251 (223, 273)	378 (316, 420)	291 (224, 348)	ns	< 0.001
**Ferritin**	NA	28.3 (18.2, 83.1)	14.4 (7.1, 37.7)	ns	-
**Hb**	139 (134, 155)	133 (125, 148)	129 (121, 154)	ns	< 0.001
**FC **	NA	1071 (346, 1389)	177 (61, 484)	ns	-

### Changes in serum biomarkers are not associated with the course of ustekinumab therapy, but differ between healthy controls and patients with IBD

**Fig 1:**
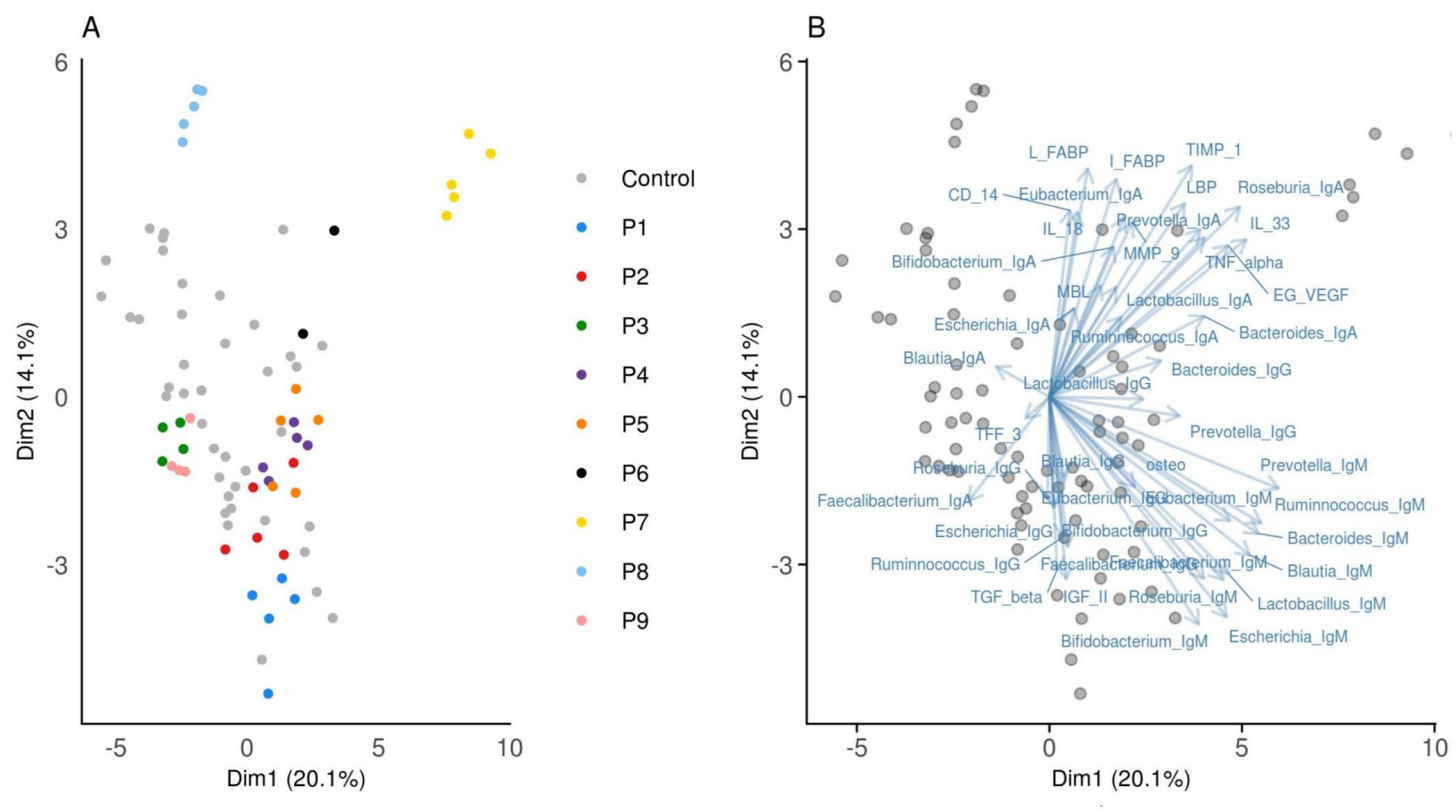
PCA of biomarker concentrations in healthy controls and patients with IBD sampled during ustekinumab treatment. Each dot represents an individual at a specific time point. A) Sample clustering along the first two PCA axes. B) Contribution of each biomarker to the sample ordination (blue arrows).

We tracked levels of 16 potential biomarkers characterizing the immune system function, mucosal layer integrity, tissue remodeling, and pattern recognition (S2 Table). Furthermore, we investigated 30 biomarkers characterizing anti-bacterial response to 10 common gut commensals during the ustekinumab treatment in patients with IBD. First, we evaluated all biomarkers together. PCA revealed that IBD samples exhibited a clear clustering based on patient´s identity, irrespective of the study phase, which point to high interindividual variability. In addition, PCA suggested a deviation of the patient’s subset from healthy controls (Fig 1A). To confirm these findings, we calculated a similarity matrix between biomarker profiles of all samples and used MDMR to evaluate group and temporal differences. We detected significant differences in biomarker profiles between healthy controls and patients with IBD (Df = 1, test stat. = 4.7100, p = 0.0160), while there was no effect of the week after treatment initiation in the subset of patients with IBD (Df = 1, test stat. = 0.1910, p = 0.7942 and Df = 4, test stat. = 3.0040, p = 0.5841 for models using the week of sample collection as a continuous or categorical predictor, respectively).

**Fig 2:**
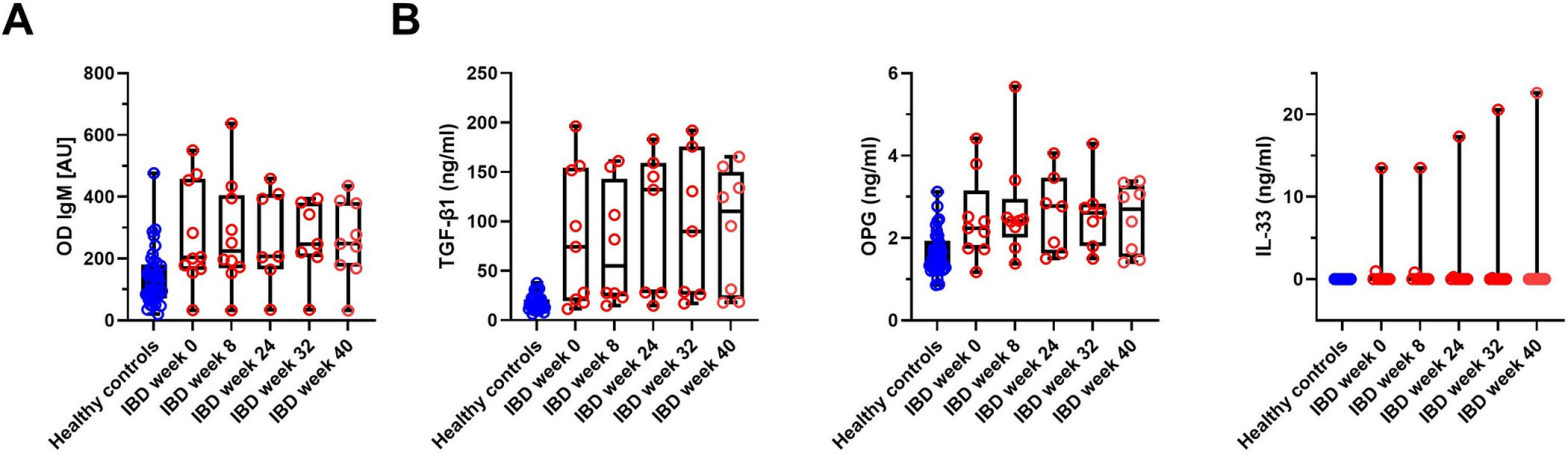
Abundance variation of biomarkers exhibiting differential expression between patients with IBD (blue) and healthy controls (red) during ustekinumab treatment. Four biomarkers identified by linear mixed effect model-based differential abundance analysis. Statistical details can be found in S3 Table. A) Serum anti-*Lactobacillus plantarum* IgM levels. B) Serum levels of TGF-β1, osteoprotegerin, and IL-33.

In Fig 1B, the relative contributions of individual biomarkers to the sample ordination are shown. To identify which of the biomarkers significantly differed between HC and patients with IBD, we performed LMM-based differential abundance analyses. These results were generally consistent with PCA (Fig 1B) and MDMR and identified 4 biomarkers with increased expression in patients with IBD compared to healthy controls (Fig 2, S4 Table). Markers including IgM antibody response to *Lactobacillus plantarum* as well as concentrations of osteoprotegerin (OPG), transforming growth factor-β (TGF-β1), and IL-33 were significantly increased in sera of patients with IBD. In addition, we looked at the temporal variation of the individual biomarkers in patients with IBD through LMM-based differential abundance analyses. To determine the best model to fit the biomarkers´ changes in time, we compared the AIC (Akaike information criterion) scores of the models tested. Null models exhibited better performance compared to more complex alternatives in most cases, suggesting no model is better than null, which means there is no association with time. Just for five biomarkers (IgG against *Bifidobacterium adolescentis*, *Roseburia intestinalis*, *Faecalibacterium prausnitzii*, *Escherichia coli* and IgM against *Roseburia intestinalis*), models including the temporal effect received substantially higher support compared to null models (i.e., ΔAIC > 3, S5 Table). However, the effect size of these temporal changes was low and none of them remained significant after multiple testing corrections (S1 Fig, S5 Table). Moreover, additional models comparing biomarkers only at week 0 and week 40 did not find any substantial changes in their concentrations (S6 Table). We conclude that in the patient group included in this study, there are no significant changes in the examined biomarkers associated with the time course of ustekinumab therapy. The only differences were identified between patients with IBD and HC.

### Ustekinumab therapy is not associated with changes in alpha or beta diversity of stool microbiota composition in patients with IBD

**Fig 3:**
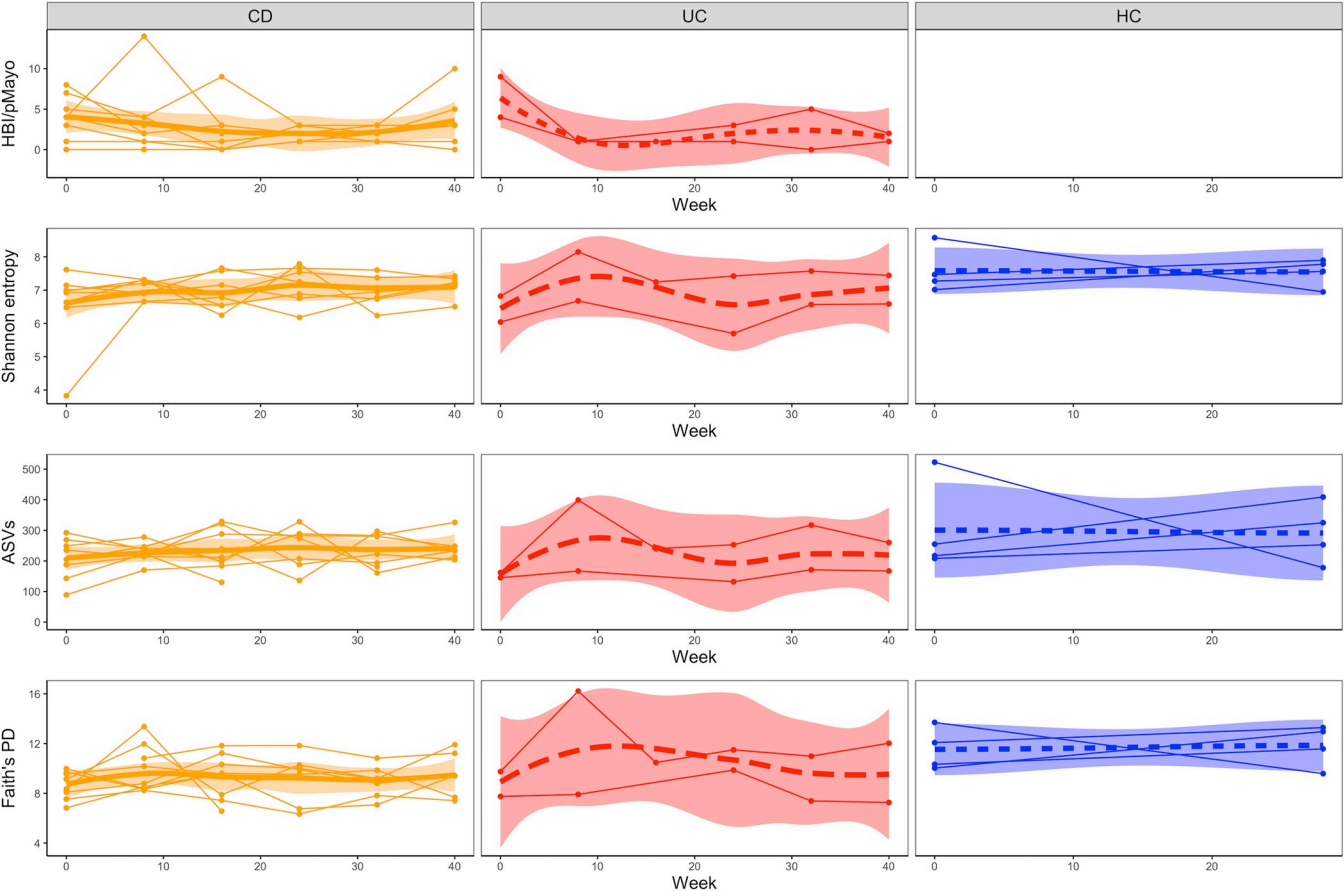
Alpha diversity variation in the stool microbiome of healthy controls and patients with Crohn’s disease and ulcerative colitis during ustekinumab treatment. The line charts show changes in disease activity scores, Shannon entropy, Chao1 diversity estimator, number of observed ASVs and Faith’s phylogenetic diversity over time for patients with Crohn’s disease, ulcerative colitis, and healthy controls. Each line represents one individual, the bold lines represent locally weighted regression with a confidence interval fill. (HBI) Harvey-Bradshaw Index, (pMayo) partial Mayo score, (PD) phylogenetic diversity.

We analyzed stool microbiome samples of patients with IBD during ustekinumab treatment and evaluated their alpha and beta diversity. To assess alpha diversity, we calculated three different metrics: observed numbers of ASVs, Shannon entropy, and Faith’s phylogenetic diversity. We plotted all these alpha diversity metrics in time for patients with CD, patients with UC, and healthy controls. We observed a more stable stool bacteriome in healthy controls than in patients with IBD (Fig 3). In all the measured metrics, only the stool bacteriome Faith´s phylogenetic diversity was decreased in patients with IBD compared to healthy controls, while other alpha diversity metrics did not differ significantly between these two groups (S7 Table, Fig 3). These data suggest that patients with IBD have a less phylogenetically diverse composition of bacteria while having comparable richness and evenness to HC (S7 and S8 Tables). We did not find any associations between the time course of therapy and alpha diversity indices in the patients’ stool microbiome (S9 Table, Fig 3). Additionally, we separated IBD patients according to their diagnosis and analyzed the differences in microbiota composition at the extreme time points. We found no differences in microbiota composition between CD and UC patients in alpha diversity neither at the study baseline (week 0) nor endpoint (week 40) (S10 Table).

**Fig 4:**
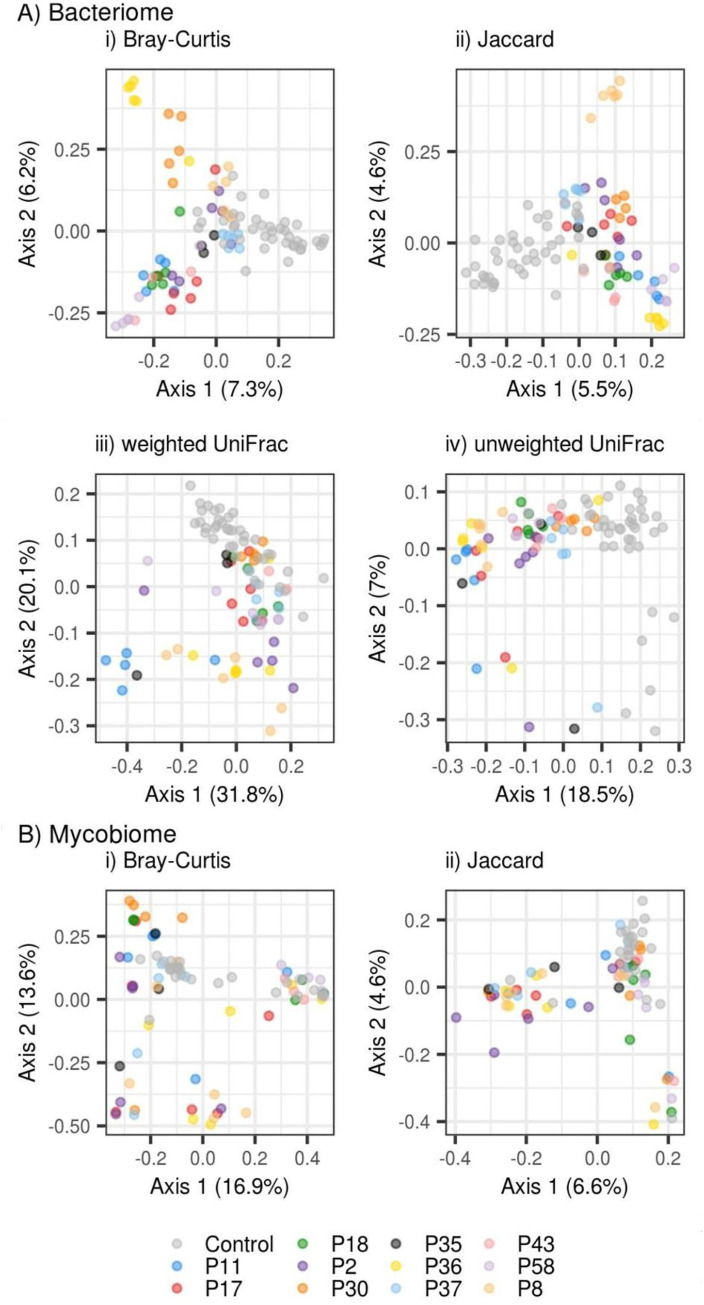
Principal coordinate analyses for stool microbiome in patients with IBD on ustekinumab therapy. Shown are A) bacteriome and B) mycobiome profiles based on different beta diversity dissimilarity measures. Beta diversity dissimilarities include Bray-Curtis, Jaccard, unweighted unique fraction metric and weighted unique fraction metric distances. Phylogenetic dissimilarities are shown only for the bacterial microbiome. UniFrac (unique fraction metric).

We also looked at the similarity of healthy controls and patients with IBD. We calculated 4 different similarity matrices, two of them considering phylogenetic relatedness (weighted and unweighted UniFrac) and two of them not (Bray-Curtis, Jaccard’s distance). Then we used these matrices in PCoAs (Fig 4). The results suggested temporal stability and high interindividual variability of the bacteriome which is a common phenomenon across gut microbiome studies. Moreover, we saw distinct clusters of patients with IBD and healthy controls when analyzing the bacteriome. To confirm these observations, we used MDMR and found substantial differences in bacterial and fungal profiles between patients with IBD and healthy controls (S11A and S12 Tables). On the other hand, we did not find support for longitudinal variation in the bacteriome or mycobiome composition of patients with IBD during ustekinumab therapy (S11B and S11C Table). The temporal effect was significant only for one model and it did not pass multiple hypothesis correction. These data indicate that in the examined patient group no changes in fecal microbial beta diversity were associated with the time course of ustekinumab therapy.

Moreover, we found no differences in beta diversity between patients with CD and UC either at baseline (S13A Table) or at the endpoint of ustekinumab therapy (S13B Table).

The results of PCoA also suggest there is a larger interindividual variability in the bacterial microbiome in patients with IBD compared to healthy controls. To test this, we calculated the centroid for each group (patients with IBD and healthy controls) and compared the distances to the individual centroid between these two groups. We found out that there is significantly higher variation in patients with IBD using weighted unique fraction metric (UniFrac), which suggests stability of the most abundant bacterial features across healthy controls and high heterogeneity among patients with IBD (S14 Table, S2 Fig).

We identified four taxa that were differentially abundant between healthy controls and patients with IBD. Specifically, we found decreased abundances of uncultured *Faecalibacterium*, *Subdoligranulum*, and *Saccharomyces cerevisiae*, and increased uncultured *Bacteroides* in the patients’ stool microbiome. Between the study baseline and endpoint, no differentially abundant taxa (both bacteria and fungi) were identified (S15 Table). Taken together, we have identified no significant longitudinal effect of ustekinumab therapy neither on alfa and beta diversity nor did we find any differentially abundant features at the study endpoint in this particular patient group.

### Ustekinumab therapy is not associated with changes in skin microbiota composition in patients with IBD

**Fig 5:**
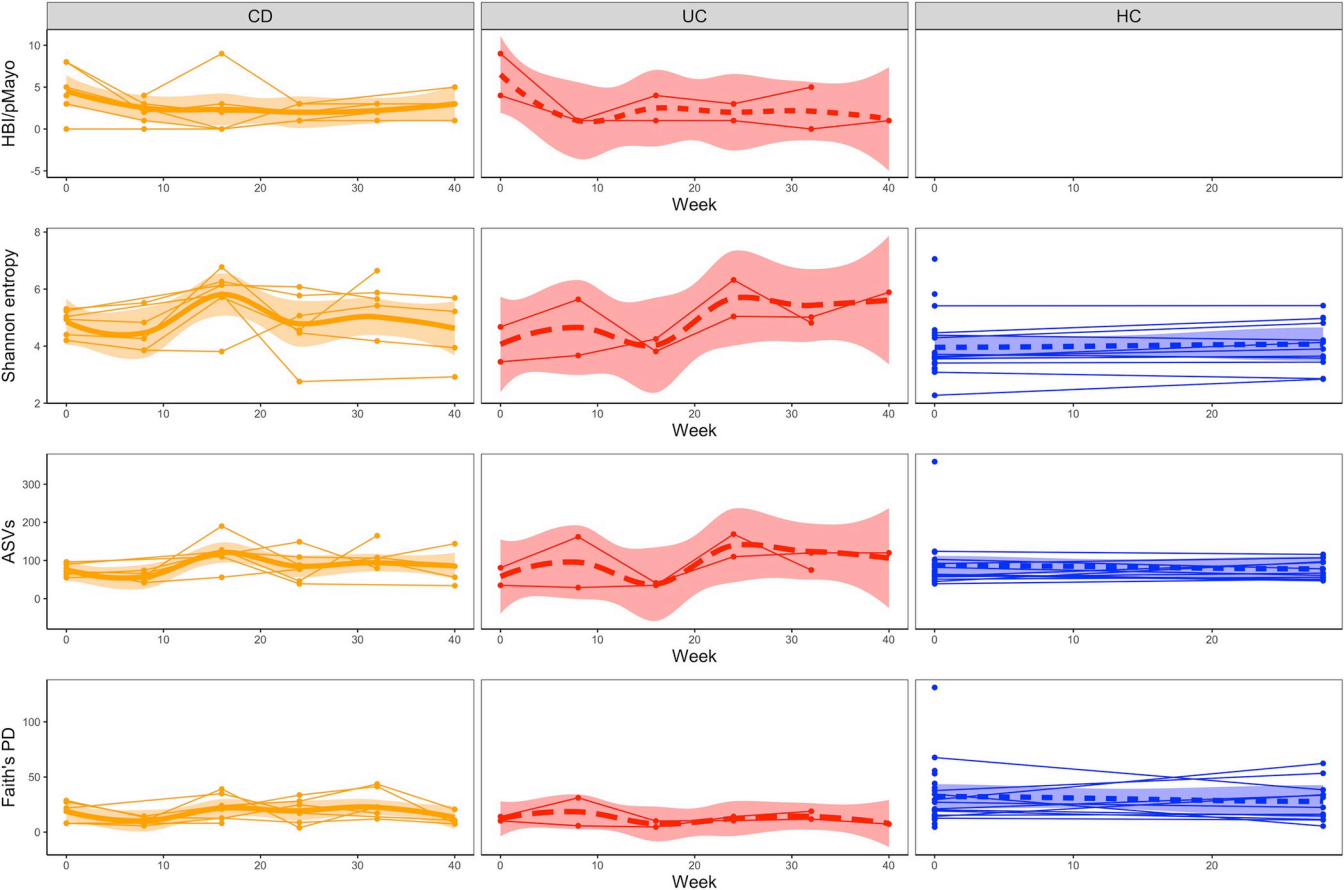
Temporal changes in different alpha diversity metrics of the skin microbiome of healthy controls, patients with Crohn’s disease, and patients with ulcerative colitis during 40 weeks of ustekinumab therapy. The line charts show changes in disease activity scores, Shannon entropy, Chao1 diversity estimator, number of observed ASVs, and Faith’s phylogenetic diversity over time for patients with Crohn’s disease, patients with ulcerative colitis, and healthy controls. Each line represents one individual, the bold lines represent locally weighted regression with a confidence interval fill. (HBI) Harvey-Bradshaw Index, (pMayo) partial Mayo score, (PD) phylogenetic diversity.

The same alpha and beta diversity metrics as in the stool microbiome were evaluated in the analysis of the skin microbiome from the retroauricular crease. Only temporal changes in the skin microbiome were investigated in this study. We have already described the comparison of the skin microbiome of healthy controls and a larger, more diverse cohort of patients with IBD, comprising also this subgroup (our unpublished data). An overview of changes in alpha diversity metrics over time is shown in Fig 5. Similar to the stool microbiome, the alpha diversities of the patients’ skin microbiome tended to fluctuate more than those of healthy controls.

To assess the effect of time, we used time as a discrete variable and compared the bacterial diversity between the study baseline (week 0) and endpoint (week 40). We found no significant changes in the alpha diversity metrics (S16A Table). Two beta diversity metrics (Jaccard’s and unweighted UniFrac distance) showed significant changes between the baseline and endpoint of the study (S17A Table), indicating a change in presence vs. absence of bacterial ASVs but not in relative abundances of dominating bacterial ASVs. Differential abundance analysis did not reveal any taxa that were significantly different in the skin microbiome of patients with IBD at baseline and endpoint of the ustekinumab therapy (S18 Table).

We also modeled time as a continuous variable (S16B Table) and found no significant temporal variation in alpha and beta diversities (S17B Table).

Overall, we did not find any continuous change in skin alpha or beta bacterial diversity but a change in beta diversity between baseline and endpoint.

### Ustekinumab therapy leads to decreased T cell response to microbiota in patients with IBD

**Fig 6:**
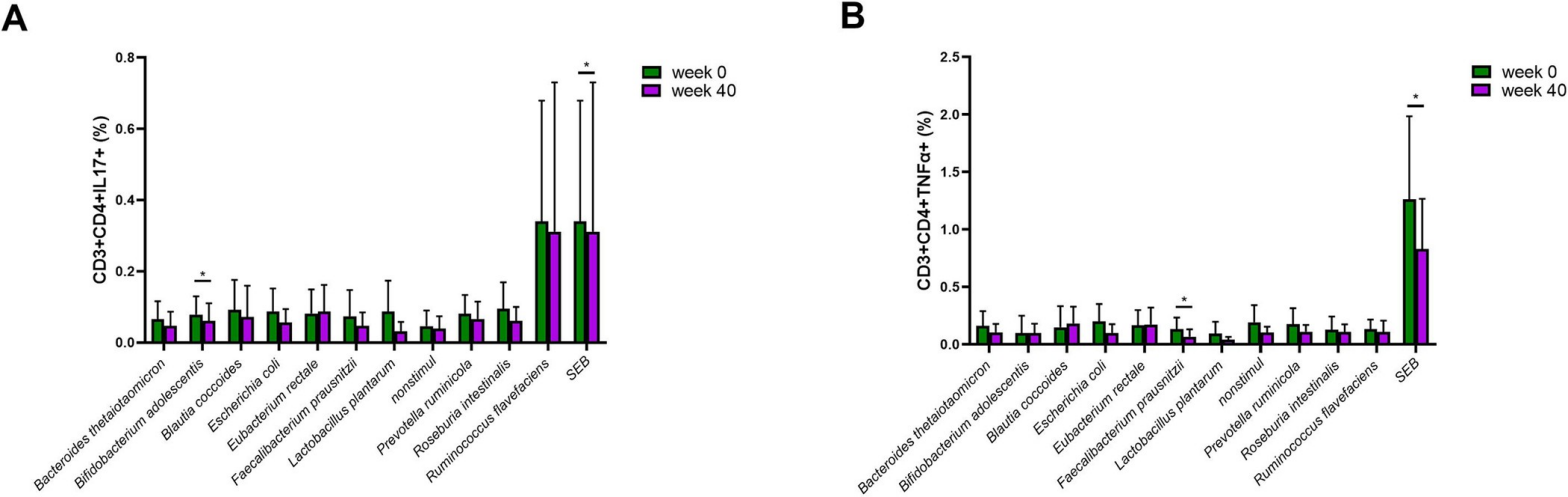
Production of IL-17 and TNF-α by circulating microbiota-reactive T cells of patients with IBD on ustekinumab therapy at baseline (week 0) and at week 40. IL-17 A) and TNF-α B) production by CD3+CD4+ T cells isolated from patients PBMC after stimulation with assorted bacterial lysates, as determined by a paired t-test. *p<0.05, IBD (n = 8), SEB (Staphylococcal enterotoxin B).

Since the immune response to microbiota might change during ustekinumab therapy, we analyzed the T cell response to microbial stimuli before the start of the therapy (week 0) and after 40 weeks. Circulating CD3+CD4+ T cells respond to gut commensal bacteria by producing several pro-inflammatory cytokines. After 40 weeks of ustekinumab therapy, their production of IL-17 and TNF-α upon stimulation with commensal bacteria lysates (*Bifidobacterium adolescentis* and *Faecalibacterium prausnitzii*), was lower (Fig 6). However, upon stimulation with the superantigen Staphylococcal enterotoxin B (SEB), our positive control, PBMCs from patients with IBD on ustekinumab therapy have a significantly lower proportion of both CD4+CD3+IL-17+ and CD3+CD4+TNFα+ T cells compared to baseline (Fig 6), suggesting that the therapy leads to a general decrease in cellular response.

## Discussion

Currently, there is an intense search for non-invasive biomarkers that could a) simplify the diagnostic procedures for IBD, b) shed more light on the mechanism of the disease and its development, and c) predict the patient’s response to specific therapies to tailor a more personalized approach to treatment. Ustekinumab represents a novel therapy, which is currently approved for several immune-mediated diseases including Crohn´s disease, ulcerative colitis, plaque psoriasis, and psoriatic arthritis. To evaluate the effect of this treatment on the stool and skin microbiome as well as the related immune response, in our pilot study we collected blood, stool, and skin samples from a small group of patients with IBD. Although, there are known differences in the pathogenesis in two main IBD phenotypes (CD and UC), IBD pathogenesis is increasingly understood as a multifactorial disorder that is initiated and exacerbated by complex influences, including genetic susceptibility, aberrant immune response and alteration in intestinal microbiota together with environmental factors [2]. To analyze these complex changes during IBD pathogenesis and obtain biomarker profile of patients undergoing ustekimumab therapy, we determined stool and skin microbiome composition longitudinally during 40 weeks of therapy. To analyze the effect of therapy on immune profile of these patients, we measured specific clinical parameters, serum levels of various biomarkers and antibodies against gut commensals, and PBMCs activity upon stimulation with specific gut commensal lysates.

We measured 46 potential biomarkers in patients’ sera and compared their levels with those of healthy controls. Generally, we observed low temporal variation during ustekinumab therapy and high interindividual variability. We identified 4 biomarkers that are differentially abundant between the two groups, which include OPG, the cytokines TGF-β1 and IL-33, and IgM antibodies against *Lactobacillus plantarum*. OPG is a decoy receptor, which is involved in the inflammatory process, bone metabolism [37], and tumorigenesis [38]. We confirmed the previously described finding that OPG is increased in patients with IBD [39,40]. It was proposed that OPG induces NF-κB activation and increases intestinal barrier permeability, which are both key features in IBD pathogenesis [41]. TGF-β1 is an immunomodulatory cytokine involved in intestinal barrier integrity, wound healing, and collagen production, and plays a key role in homeostasis maintenance [42]. This study is in line with several other studies reporting increased levels of TGF-β1 in patients with IBD [43,44] while others report increased levels in patients with successful anti-TNF treatment, suggesting an active suppression of the inflammatory response and tissue repair [45]. IL-33 is another immunomodulatory cytokine, whose role probably depends on the type of inflammatory context. Some studies show its protective function in restoring goblet cell numbers and switching macrophage phenotype from M1 (pro-inflammatory) to M2 (wound healing) [46]; other studies propose that IL-33 and its ligand ST2 are involved in IBD pathogenesis by increasing intestinal barrier permeability [47]. In our patient cohort, IL-33 was not detectable in the majority of patients similarly to a study by Beltran et al. [48]. Nevertheless, we detected high levels of IL-33 in three individuals. Unfortunately, we did not identify any common features or consistent changes in disease severity in these individuals that would indicate tissue damage or repair.

It is now commonly believed that IBD arises from disruption of the protective intestinal barrier and subsequent exposure of the underlying immune system components to bacterial antigens. Here, we have identified an increased IgM antibody response against *Lactobacillus plantarum* in patients with IBD. Although we did not find lactobacilli to be differentially abundant between HC and patients with IBD in our cohort, lactobacilli have been previously reported to be increased in patients with both CD and UC [49,50]. An antigen from bacterial flagella, which is shared by many bacteria, has been shown to elicit a strong IgG response in patients with CD [51]. However, it is worth noting that even healthy individuals have homeostatic levels of anti-commensal IgG and IgM [16,52]. Collectively, the serum biomarkers differed only between patients and healthy controls, with no associations with the time course of ustekinumab therapy.

Becker et al. proposed that bacteria in the terminal ileum can induce a constitutive expression of p40, a common subunit of IL-12 and IL-23, in a subset of lamina propria dendritic cells [53]. Therefore, we asked whether treatment with monoclonal antibodies targeting the p40 subunit protects from a bacteria-induced inflammation and whether this therapeutic blockage would result in a microbiome composition shift. In our patient group, we revealed significant differences between the stool microbiome of healthy controls and patients with IBD. We observed a significantly decreased Faith’s phylogenetic diversity in patients with IBD compared to healthy controls. It should be noted that similar differences in multiple diversity metrics were reported by others regardless of therapy [54,55]. The low phylogenetic diversity in patients with IBD suggests a dysbiotic state of their fecal microbiota, which was not significantly improved after 40 weeks of therapy. We did not find any differences in other diversity metrics. That could be explained by the fact that the majority of recruited patients suffered from CD localized to the terminal ileum or ileocolon, whereas fecal microbiota is more representative of the colonic community. Taken together, the stool diversity data demonstrate that healthy controls have a more phylogenetically diverse set of bacteria, with less variation between individuals, consistent with the idea of a core microbiome [56,57]. Moreover, we identified three bacteria and one fungus as differently abundant, with uncultured *Bacteroides* being the only taxon increased in patients with IBD. *Bacteroides* species are common gut commensals that can utilize the complex glycans available in the intestine, but under certain conditions can also promote intestinal pathology. *B*. *thetaiotaomicron* is capable of digesting the mucin O-glycans formulating the protective layer between the intestinal lumen and epithelium and could therefore contribute to IBD pathogenesis [58]. Other *Bacteroides* species were also found to be associated with IBD, like *Bacteroides fragilis* [59] and *Bacteroides vulgatus* [60]. Moreover, we detected decreased levels of the bacterial genera *Faecalibacterium* and *Subdoligranulum* in patients with IBD. *Faecalibacterium* exhibits anti-inflammatory properties and is often reported to be decreased in patients with IBD [61,62]. *Subdoligranulum*, a close phylogenetic relative of *Faecalibacterium*, was also repeatedly reported to be associated with a healthy phenotype [63–65], which is consistent with our data. In the fungal microbiome, we identified *Saccharomyces cerevisiae* as decreased in patients with IBD. However, fungi detected from stool specimens may not be stable residents of the intestinal environment and can be just passing through the gut [66]. Furthermore, their presence is heavily influenced by diet [67,68]. Nevertheless, an increased abundance of *S*. *cerevisiae* has been reported in pediatric patients with CD, suggesting that the immunogenic properties of *S*. *cerevisiae* are strain-specific [69].

The more diverse microbiome of the healthy intestine after successful treatment may be behind recent finding describing changes in the composition of the microbiota in IBD patients receiving biologic therapy [28]. Interestingly, Doherty *et al*. 2018 showed that α-diversity increased over time in patients who respond to ustekinumab therapy, in contrast to patients who did not. They identified specific microbial associations, such as higher abundance of *Faecalibacterium*, *Bacteroides*, and *Ruminococcus* species at baseline, which were subsequently associated with disease remission, and higher abundance of *Faecalibacterium*, Ruminococcaceae, and *Blautia*, which were associated with response to therapy [70]. These species were associated with a healthy microbiome [71].

It is also important to note that most patients treated with ustekinumab received conventional therapy or other biologics, such as anti-TNF inhibitors, prior to this treatment, which could partially alter the composition of the microbiota [72–74]. To date, no study compared the changes in the microbiota composition in drug naïve IBD patients or in IBD patients with various treatment strategies. Further, more detailed studies are also needed due to possible modification of the treatment efficacy by the microbiome itself similarly as in e.g. cancer immunotherapy [75].

About 5% of patients with IBD treated with anti-TNF drugs develop an adverse event such as skin lesions, which are filled with Th1 and Th17 cells producing interferon-γ, IL-17A, and IL-22. These adverse effects can resolve on ustekinumab therapy [76]. The skin lesions probably arise from an imbalance in the cytokine milieu and can promote the development of paradoxical psoriasis [77,78]. Previous studies from our group indicate that the skin microbiome of patients with IBD and psoriatic patients differs from that of healthy controls (our unpublished data and [26]. Therefore, we decided to determine whether patients with IBD experience changes in the skin microbiome during treatment with ustekinumab. We found a significant beta diversity change between the therapy baseline and endpoint. Two different beta diversity metrics, which are not dependent on abundance, suggest that rare taxa could play a role in this scenario. This is an interesting finding, which must be however further validated in a larger cohort of patients. It is worth noting that none of the patients in our cohort developed an adverse skin reaction and that 5 out of the 9 patients who provided skin swabs have a history of anti-TNF treatment. Given that we did not find any temporal trend in beta diversity when sampling at multiple time points, we conclude that we do not have sufficient evidence to prove the association with ustekinumab treatment. Additionally, we observed no significant changes in alpha diversity or abundance of particular taxa in time.

IBD pathogenesis is tightly connected with altered microbiota composition and its interactions with resident T cells. During the inflammation that accompanies IBD pathogenesis, T cells are polarized to Th1 or Th17 subsets, which are characterized by the production of cytokines, such as IFN-γ, TNF-α, and IL-17 [79]. These cytokines can possess both pro-inflammatory and anti-inflammatory properties [80,81]. Here, we analyzed the impact of ustekinumab therapy on the presence of CD3+CD4+IL-17+ and CD3+CD4+TNF-α+ T cells in patients with IBD and their response to specific intestinal commensal bacteria common in the Czech population. In our previous study, we challenged PBMC of patients with IBD on anti-TNF therapy and observed the upregulation of IL-17 production upon stimulation by *E*. *coli* or *Blautia coccoides* [28]. In contrast, in the present study, we detected a downregulation of CD3+CD4+IL-17+ and CD3+CD4+TNF-α+ T cells irrespective of the stimulus. This likely indicates a general decrease in the cellular immune response. This could be a direct outcome of the therapy, as IL-17 is downstream of IL-23 signaling. This is also supported by the fact that we saw a significant decrease after a non-specific stimulation with the superantigen SEB, which we used as a positive control. Nevertheless, a remarkable decrease in IL-17 was detected upon stimulation by *Bifidobacterium adolescentis*. This finding was to be expected since several strains of *Bifidobacterium* are able to downregulate IL-17 production during intestinal inflammation [82,83]. Moreover, the presence of *B*. *adolescentis* was described to be decreased in patients with IBD compared to healthy controls [83]. An increasing number of studies have shown that *F*. *prausnitzii* is significantly decreased in patients with acute IBD and its abundance correlates with disease activity [84–86]. We found a significant downregulation of the expression of CD3+CD4+TNF-α+ T cells upon *F*. *prausnitzii* lysate stimulation in patients with IBD undergoing ustekinumab therapy. Our findings thus provide further evidence of the anti-inflammatory effect of *F*. *prausnitzii*. In summary, we observed a downregulation of CD3+CD4+IL-17+ and CD3+CD4+TNF-α+ T cells upon specific and non-specific bacterial antigen stimulation. However, we cannot rule out that the drop in T cell response was generalized and was caused by the blockade of IL-12/23 itself rather than linked to specific antigenic stimuli.

To conclude, this pilot study gives an exhaustive description of multiple serum, fecal and skin biomarkers, which were measured longitudinally in a small group of patients with IBD during 40 weeks of treatment with ustekinumab. We took advantage of this unique longitudinal design together with complex analyses allowing to reveal some associations of skin beta diversity and T cell response with the course of therapy. More in-depth studies need to be conducted to confirm these findings, but the significance of this study lies in presenting initial information and ideas others can expand on.

## Supporting information

S1 Fig Temporal variation of four biomarkers, where the null model received a considerably lower support than a more complex model version estimating the temporal effect (ΔAIC > 3). Note that none of these effects remained significant after correction for multiple testing. Here we show actual concentrations and model predictions ± 95% confidence intervals.
(DOCX)

S2 Fig Individual variation in microbiota composition in healthy controls and patients with inflammatory bowel diseases sampled during ustekinumab treatment. Boxplots depict the variance in the microbiota composition within the group of healthy controls and patients with IBD by showing the distances to the centroid of each group. Comparisons were done using linear mixed effect models.
(DOCX)

S1 Table Summary of sample collection counts. Skin, stool, and serum samples were collected. The number of samples collected per individual for patients with IBD and healthy controls is displayed.
(DOCX)

S2 Table A list of 16 ELISA kits used for biomarker detection. (DOCX)

S3 Table Clinical characteristics of patients with Crohn’s disease (CD) and Ulcerative colitis (UC) in our study cohort. UST (ustekinumab), IFX (infliximab), VEDO (vedolizumab), ADA (adalimumab).
(DOCX)

S4 Table Biomarker abundance differences between healthy controls and patients with IBD based on linear mixed effect models. Mean, SE (standard error), DF (degrees of freedom), test statistic (Χ^2^) and corresponding *p* values are shown. *Q*-value method was used for multiple testing corrections. IBD (patients with inflammatory bowel disease), HC (healthy controls).
(DOCX)

S5 Table Temporal variation of serum biomarker concentrations in patients with IBD treated with ustekinumab. Longitudinal changes were modeled via linear mixed effect models, where the temporal variation was fitted as a linear continuous predictor, quadratic polynomial term, piecewise polynomials term fitted via B splines or as a categorial predictor. Performance of alternative models was assessed based on Akaike information criterion difference (ΔAIC) between the best fitting model and corresponding AIC weights. *P* values were derived based on the deviance change between the null model and the best fitting non-null model assuming its χ^2^ distribution. *Q* value method for estimating false discovery rate was used to correct for multiple hypothesis testing.
(DOCX)

S6 Table Change in serum biomarker concentrations in patients with IBD treated with ustekinumab between week 0 and week 40. Mean values and their standard deviations (in parentheses) are shown. P values were derived on the basis of the likelihood ratio test, which assumes a χ2 distribution of deviance changes. The Q-value method for estimating the false discovery rate was used to correct for multiple hypothesis testing.
(DOCX)

S7 Table Results of linear mixed effect models testing for differences in alpha diversity metrics between the stool microbiome of patients with IBD and healthy controls. Values for test statistics (Χ2), associated degrees of freedom (DF) and resulting p values are shown. ASV (amplicon sequence variants).
(DOCX)

S8 Table Results of non-parametric Kruskal-Wallis test comparing differences in alpha diversity metrics between the stool microbiome of patients with IBD and healthy controls A) at baseline (week 0) and B) endpoint (week 40). Values for test statistics (H), associated degrees of freedom (DF) and resulting *p* and *q* values are shown. ASV (amplicon sequence variants), HC (healthy controls), IBD (inflammatory bowel disease).
(DOCX)

S9 Table Temporal variation of stool microbial alpha diversity of patients with IBD treated with ustekinumab. Longitudinal changes were modeled via linear mixed effect models, where the temporal variation was fitted as linear continuous predictor, quadratic polynomial term, piecewise polynomials term fitted via B splines or as a categorial predictor. Performance of alternative models was assessed based on Akaike information criterion difference (ΔAIC) between the best fitting model and corresponding AIC weights. *P* values were derived based on the deviance change between the null model and the best fitting non-null model assuming its χ^2^ distribution. *Q* value method for estimating false discovery rate was used to correct for multiple hypothesis testing. PD (phylogenetic diversity), ASV (amplicon sequence variants).
(DOCX)

S10 Table Results of non-parametric Kruskal-Wallis test comparing differences in alpha diversity metrics between the stool microbiome of patients with CD and UC A) at baseline (week 0) and B) endpoint (week 40). Values for test statistics (H), associated degrees of freedom (DF) and resulting *p* and *q* values are shown. ASV (amplicon sequence variants), CD (Crohn’s disease), UC (ulcerative colitis).
(DOCX)

S11 Table Results of multivariate distance matrix regression (MDMR) testing for variation in stool microbiome composition of patients with IBD during ustekinumab treatment. MDMR testing differences in community composition **A)** between IBD patients and healthy controls. The week of sample collection was fitted as a **B)** categorial or **C)** continuous predictor. Values for test statistics, associated degrees of freedom (DF) and resulting *p* values are shown. IBD (patients with inflammatory bowel disease), HC (healthy controls), w (weighted), u (unweighted).
(DOCX)

S12 Table Microbial beta diversity variability between a group of healthy controls (HC) and patients with IBD **A**) at the baseline (week 0), **B**) at the endpoint (week 40) was assessed with permutational tests PERMANOVA and PERMDISP using 1000 permutations. Values for test statistics, *p* values, adjusted *q* values and degrees of freedom (DF) are reported.
(DOCX)

S13 Table Microbial beta diversity variability between a group of CD patients and UC patients **A**) at the baseline (week 0), **B**) at the endpoint (week 40) was assessed with permutational tests PERMANOVA and PERMDISP using 1000 permutations. Values for test statistics, p values, adjusted q values and degrees of freedom (DF) are reported.
(DOCX)

S14 Table Variability within the group of healthy controls and patients with IBD. To compare individual variation between IBD patients and healthy controls, distances to group-specific centroids were calculated using the vegan::betadisper() function in R and included as the response in a mixed model with group identity as a predictor and individual identity as random intercepts. Separate models were fitted for different types of dissimilarity matrices describing beta diversity in bacterial and fungal profiles. Significance was assessed based on likelihood ratio tests assuming a χ^2^-distribution of deviance changes, followed by the *q*-value multiple testing correction method.
(DOCX)

S15 Table Differentially abundant taxa from stool of patients with IBD and healthy controls. The table shows differentially abundant taxa determined by ANCOM2.1 between **A)** patients with inflammatory bowel disease (IBD) and healthy controls (HC) and between patients at **B)** baseline (week 0) and at the endpoint (week 40) of the study. W-statistics and centred log ratios (CLR) are shown. Wmax was 117 and 32 for the whole cohort including HC and IBD patients bacteriome and mycobiome, respectively (A). Wmax was 83 and 16 for IBD patients’ bacteriome and mycobiome, respectively (B). A 0.8W cut-off was chosen. uncultured (unctl.), structural zero (str. zero).
(DOCX)

S16 Table Temporal variation of microbial skin alpha diversity of patients with IBD treated with ustekinumab. None of the alpha diversity metrics show a significant difference in the skin microbiome of IBD patients between the baseline (week 0) and endpoint (week 40) of the ustekinumab therapy **A)**. Same results were obtained when week was fitted as a continuous variable **B)**. Values for test statistics, associated degrees of freedom (DF), and resulting *p* values and *q* values with correction for multiple hypothesis testing (false discovery rate) are shown.
(DOCX)

S17 Table Results of multivariate distance matrix regression (MDMR) testing for variation in the skin microbiome composition of patients with IBD during ustekinumab treatment. MDMR testing differences in community composition between week 0 and week 40 A) or using week as a continuous variable B). Values for test statistics, associated degrees of freedom (DF) and resulting *p* values and *q* values with correction for multiple hypothesis testing (false discovery rate) are shown.
(DOCX)

S18 Table Taxa found on the skin of IBD patients that are differentially abundant between the baseline and endpoint of the study. The table shows differentially abundant taxa determined by ANCOM2.1 between the baseline (week 0) and the endpoint (week 40) of the study on the skin of patients with IBD treated with ustekinumab. W-statistics and centred log ratios (CLR) are shown. Wmax was 23 and a 0.8W cut-off was chosen. Structural zero (str. zero).
(DOCX)
