# Supplementary material for: Association between ustekinumab therapy and changes in specific anti-microbial response, serum biomarkers, and microbiota composition in patients with IBD: A pilot study
Source: PLoS One. 2022 Dec 30;17(12):e0277576. doi: 10.1371/journal.pone.0277576 (PMC9803183; doi:10.1371/journal.pone.0277576)
Supplement: S1 Fig — Note that none of these effects remained significant after correction for multiple testing. Here we show actual concentrations and model predictions ± 95% confidence intervals. (DOCX) [file pone.0277576.s001.docx]

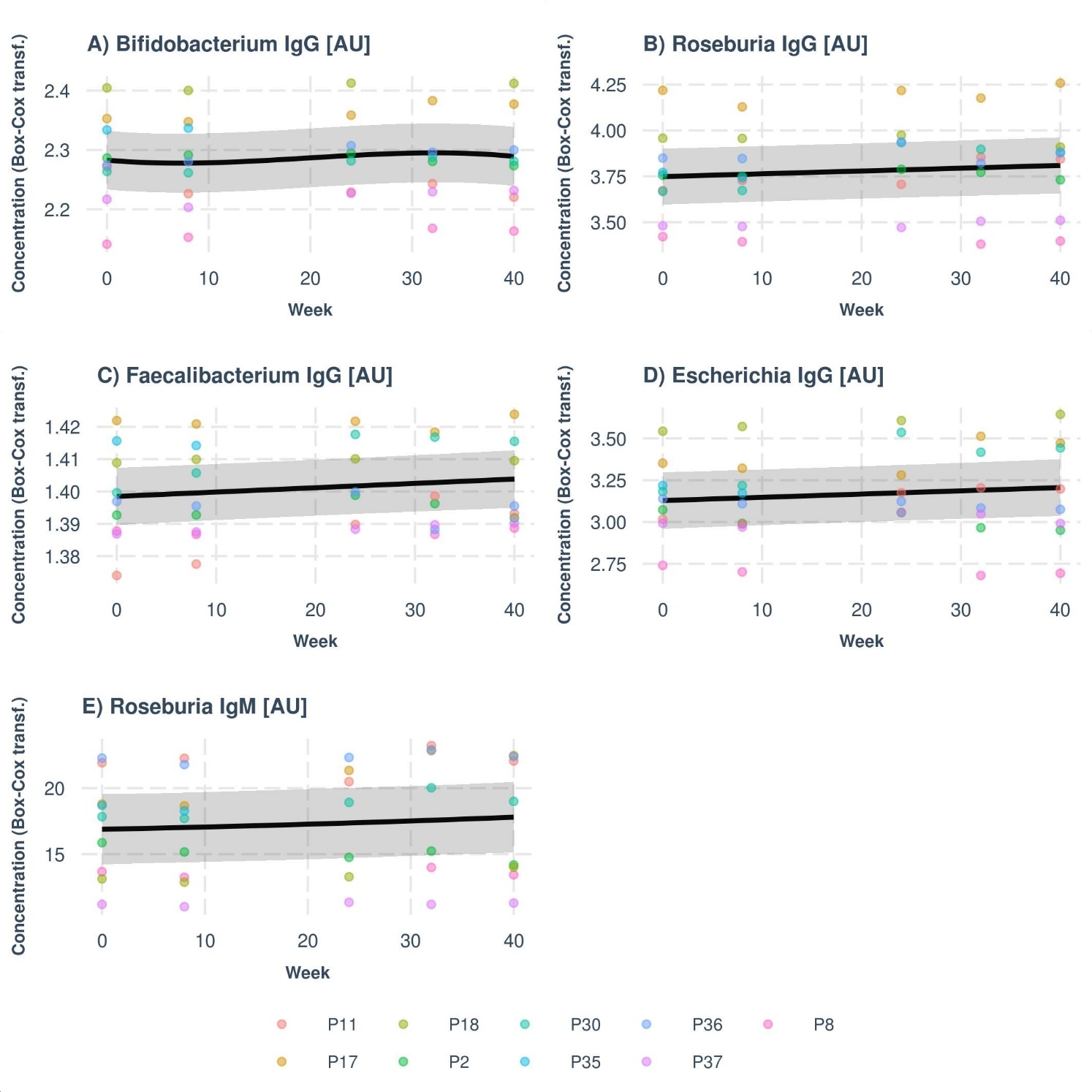


**Supplementary Figure 1.** Temporal variation of four biomarkers, where the null model received a considerably lower support than a more complex model version estimating the temporal effect (ΔAIC > 3). Note that none of these effects remained significant after correction for multiple testing. Here we show actual concentrations and model predictions ± 95% confidence intervals. AU (arbitrary units).
