# Supplementary material for: Association between ustekinumab therapy and changes in specific anti-microbial response, serum biomarkers, and microbiota composition in patients with IBD: A pilot study
Source: PLoS One. 2022 Dec 30;17(12):e0277576. doi: 10.1371/journal.pone.0277576 (PMC9803183; doi:10.1371/journal.pone.0277576)
Supplement: S2 Fig — Boxplots depict the variance in the microbiota composition within the group of healthy controls and patients with IBD by showing the distances to the centroid of each group. Comparisons were done using linear mixed effect models. (DOCX) [file pone.0277576.s002.docx]

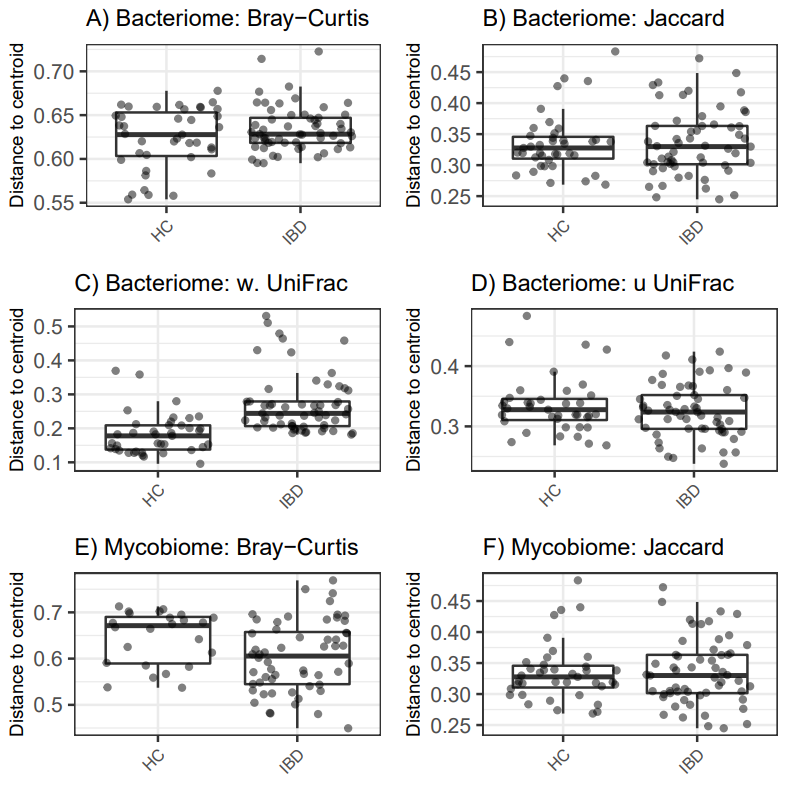


**Supplementary Figure 2:** Individual variation in microbiota composition in healthy controls and patients with inflammatory bowel diseases sampled during ustekinumab treatment. Boxplots depict the variance in the microbiota composition within the group of healthy controls and patients with IBD by showing the distances to the centroid of each group. Comparisons were done using linear mixed effect models. HC (healthy controls), IBD (inflammatory bowel disease), w (weighted), u (unweighted).
