# Supplementary material for: Association between ustekinumab therapy and changes in specific anti-microbial response, serum biomarkers, and microbiota composition in patients with IBD: A pilot study
Source: PLoS One. 2022 Dec 30;17(12):e0277576. doi: 10.1371/journal.pone.0277576 (PMC9803183; doi:10.1371/journal.pone.0277576)
Supplement: S1 Table — Skin, stool, and serum samples were collected. The number of samples collected per individual for patients with IBD and healthy controls is displayed. (DOCX) [file pone.0277576.s003.docx]

**Supplementary Table 1**: Summary of sample collection counts. Skin, stool, and serum samples were collected. The number of samples collected per individual patient with IBD and healthy control is displayed.

| **Patient ID** | **skin samples** | **stool samples** | **serum samples** |
| --- | --- | --- | --- |
| P1 | 6 | 6 | 5 |
| P2 | 4 | 6 | 4 |
| P3 | 5 | 5 | 5 |
| P4 | 6 | 6 | 5 |
| P5 | 5 | 5 | 4 |
| P6 | 6 | 5 | 5 |
| P7 |  | 3 | 2 |
| P8 | 4 | 6 | 5 |
| P9 | 6 | 6 | 5 |
| P10 | 2 | 3 |  |
| P11 |  | 6 |  |
| **Total number of samples collected** | **44** | **57** | **40** |
| **Total number of individuals** | **9** | **11** | **9** |
| **Healthy control ID** | **skin samples** | **stool samples** | **serum samples** |
| HC1 | 2 | 2 | 1 |
| HC2 | 2 | 1 | 1 |
| HC3 | 1 | 1 | 1 |
| HC4 | 2 | 1 | 1 |
| HC5 |  | 1 | 1 |
| HC6 |  | 1 | 1 |
| HC7 | 1 | 1 | 1 |
| HC8 | 2 | 1 | 1 |
| HC9 |  | 2 | 1 |
| HC10 | 2 | 1 | 1 |
| HC11 | 2 | 1 | 1 |
| HC12 |  | 1 | 1 |
| HC13 |  | 1 | 1 |
| HC14 |  | 2 | 1 |
| HC15 | 2 | 1 | 1 |
| HC16 | 2 | 1 | 1 |
| HC17 |  | 1 | 1 |
| HC18 |  | 1 | 1 |
| HC19 | 2 | 1 | 1 |
| HC20 | 1 | 1 | 1 |
| HC21 |  | 1 | 1 |
| HC22 | 1 | 1 | 1 |
| HC23 |  | 1 | 1 |
| HC24 |  | 1 | 1 |
| HC25 |  |  | 1 |
| HC26 | 1 | 1 | 1 |
| HC27 | 1 |  | 1 |
| HC28 | 1 | 1 | 1 |
| HC29 |  | 2 | 1 |
| HC30 | 1 | 1 | 1 |
| HC31 |  | 1 | 1 |
| HC32 | 2 | 1 | 1 |
| HC33 |  | 1 | 1 |
| HC34 |  | 1 |  |
| HC35 |  | 1 |  |
| HC36 |  | 1 | 1 |
| HC37 | 1 | 1 |  |
| HC38 | 1 | 1 | 1 |
| HC39 | 2 | 1 | 1 |
| HC40 |  | 1 | 1 |
| HC41 | 1 | 1 | 1 |
| **Total number of samples collected** | **33** | **43** | **38** |
| **Total number of individuals** | **22** | **39** | **38** |
