# Supplementary material for: Association between ustekinumab therapy and changes in specific anti-microbial response, serum biomarkers, and microbiota composition in patients with IBD: A pilot study
Source: PLoS One. 2022 Dec 30;17(12):e0277576. doi: 10.1371/journal.pone.0277576 (PMC9803183; doi:10.1371/journal.pone.0277576)
Supplement: S2 Table — (DOCX) [file pone.0277576.s004.docx]

**Supplementary Table 2**: A list of 16 ELISA kits used for biomarker detection.

| **Biomarker** | **Abbreviation** | **Manufacturer** | **Cat. No** |
| --- | --- | --- | --- |
| Endocrine-Gland-derived Vascular Endothelial Growth Factor | EG-VEGF | R&D systems | DY1209 |
| Osteoprotegerin | OPG | R&D systems | DY805 |
| Insulin-like Growth Factor 2 | IGF2 | R&D systems | DY292 |
| Transforming Growth Factor-β1 | TGF-β1 | R&D systems | DY240 |
| Matrix Metalloproteinase 9 | MMP-9 | R&D systems | DY911 |
| Matrix Metalloproteinase 14 | MMP-14 | R&D systems | DY918 |
| Tissue Inhibitor of Metalloproteinases 1 | TIMP-1 | R&D systems | DY970 |
| Mannan-Binding Lectin | MBL | R&D systems | DY2307 |
| Soluble CD14 | CD14 | R&D systems | DY383 |
| Lipopolysaccharide-Binding Protein | LBP | R&D systems | DY870 |
| Trefoil Factor – 3 | TFF-3 | R&D systems | DY4407 |
| Tumor necrosis factor | TNF-α | R&D systems | DY210 |
| Intestinal fatty acid-binding protein | I-FABP | R&D systems | DY3078 |
| Liver fatty acid-binding protein | L-FABP | HyCult Biotech | HK404 |
| Interleukin 33 | IL-33 | R&D systems | DY3625 |
| Interleukin 18 | IL-18 | R&D systems | DY318 |
