# Supplementary material for: Association between ustekinumab therapy and changes in specific anti-microbial response, serum biomarkers, and microbiota composition in patients with IBD: A pilot study
Source: PLoS One. 2022 Dec 30;17(12):e0277576. doi: 10.1371/journal.pone.0277576 (PMC9803183; doi:10.1371/journal.pone.0277576)
Supplement: S3 Table — UST (ustekinumab), IFX (infliximab), VEDO (vedolizumab), ADA (adalimumab). (DOCX) [file pone.0277576.s005.docx]

**Supplementary Table 3**: Clinical characteristics of patients with Crohn’s disease (CD) and ulcerative colitis (UC) in our study cohort. UST (ustekinumab), IFX (infliximab), VEDO (vedolizumab), ADA (adalimumab).

| **Patient ID** | **Gender** | **Diagnosis** | **Drug** | **Brand** | **Disease duration (years)** | **Age at diagnosis (years)** | **Montreal Clasification** | **HB index at baseline** | **HB index at week 40** | **Mayo index at baseline** | **Mayo index at week 40** | **CRP (mg/L) at baseline** | **CRP (mg/L) at week 40** | **Response to ustekinumab at week 40** | **Other therapy before ustekinumab** | **Previous other biological theraphy** |
| --- | --- | --- | --- | --- | --- | --- | --- | --- | --- | --- | --- | --- | --- | --- | --- | --- |
| P1 | Male | CD | UST | Stelara | 11 | 22 | A2B1L3 | 0 | 1 |  |  | 13.5 | 2.3 | yes | yes | IFX |
| P2 | Female | CD | UST | Stelara | 10 | 32 | A2B3L1 | 7 | 10 |  |  | 15.3 | 3 | yes | yes | IFX |
| P3 | Female | UC | UST | Stelara | 10 | 25 | E2 |  |  | 12 | 2 | 0.7 | 0.4 | yes | yes | IFX, VEDO |
| P4 | Female | CD | UST | Stelara | 24 | 37 | A2B2L3 | 8 | 3 |  |  | 3.8 | 2 | yes | yes | none |
| P5 | Male | CD | UST | Stelara | 1 | 43 | A2B3L1 | 3 | 3 |  |  | 5.8 | 6.8 | yes | yes | none |
| P6 | Female | CD | UST | Stelara | 8 | 39 | A2B3L1 | 8 | 3 |  |  | 3.7 | 4 | yes | yes | IFX |
| P7 | Male | CD | UST | Stelara | 12 | 13 | A1B3L1 | 4 | 1 |  |  | 8.1 | 9.9 | yes | yes | IFX |
| P8 | Male | CD | UST | Stelara | 14 | 27 | A2B3L1 | 5 | 5 |  |  | 6.4 | 5 | no | yes | IFX, ADA |
| P9 | Male | UC | UST | Stelara | 15 | 49 | E3 |  |  | 4 | 1 | 10.3 | 2.3 | yes | yes | VEDO |
| P10 | Female | CD | UST | Stelara | 1 | 55 | A2B1L1 | 4 | NA |  |  | 8.8 | NA | NA | yes | none |
| P11 | Male | CD | UST | Stelara | 5 | 30 | A2B3L3 | 1 | 0 |  |  | 2.8 | 4.3 | yes | yes | ADA |
