# Supplementary material for: Association between ustekinumab therapy and changes in specific anti-microbial response, serum biomarkers, and microbiota composition in patients with IBD: A pilot study
Source: PLoS One. 2022 Dec 30;17(12):e0277576. doi: 10.1371/journal.pone.0277576 (PMC9803183; doi:10.1371/journal.pone.0277576)
Supplement: S4 Table — Mean, SE (standard error), DF (degrees of freedom), test statistic (Χ2) and corresponding p values are shown. Q-value method was used for multiple testing corrections. IBD (patients with inflammatory bowel disease), HC (healthy controls). (DOCX) [file pone.0277576.s006.docx]

**Supplementary Table 4**: Biomarker abundance differences between healthy controls and patients with IBD based on linear mixed effect models. Mean, SE (standard error), DF (degrees of freedom), test statistic (Χ^2^) and corresponding *p* values are shown. *Q*-value method was used for multiple testing corrections. IBD (patients with inflammatory bowel disease), HC (healthy controls).

| **Biomarker** | **Mean HC** | **SE HC** | **Mean IBD** | **SE IBD** | **DF** | **Χ^2^** | ***p* value** | ***q* value** |
| --- | --- | --- | --- | --- | --- | --- | --- | --- |
| IgA *Lactobacillus* | 491.574 | 64.231 | 451.180 | 34.202 | 1 | 0.409 | 0.523 | 0.752 |
| IgA *Bifidobacterium* | 664.639 | 137.787 | 544.611 | 41.941 | 1 | 0.357 | 0.550 | 0.752 |
| IgA *Blautia* | 332.879 | 45.857 | 845.543 | 144.801 | 1 | 6.908 | 0.009 | 0.057 |
| IgA *Roseburia* | 424.136 | 57.657 | 797.854 | 196.090 | 1 | 0.367 | 0.545 | 0.752 |
| IgA *Faecalibacterium* | 422.415 | 80.766 | 291.092 | 44.968 | 1 | 4.222 | 0.040 | 0.150 |
| IgA *Bacteroides* | 230.915 | 34.887 | 324.212 | 30.972 | 1 | 3.460 | 0.063 | 0.202 |
| IgA *Escherichia* | 233.882 | 31.261 | 223.226 | 18.935 | 1 | 0.202 | 0.653 | 0.816 |
| IgA *Prevotella* | 577.831 | 116.189 | 981.686 | 120.757 | 1 | 4.086 | 0.043 | 0.150 |
| IgA *Ruminnococcus* | 396.522 | 66.411 | 470.684 | 37.545 | 1 | 2.031 | 0.154 | 0.345 |
| IgA *Eubacterium* | 673.134 | 97.831 | 966.087 | 191.427 | 1 | 0.485 | 0.486 | 0.752 |
| IgG *Lactobacillus* | 772.338 | 119.741 | 470.186 | 55.810 | 1 | 0.370 | 0.543 | 0.752 |
| IgG *Bifidobacterium* | 1095.875 | 166.136 | 872.637 | 174.814 | 1 | 0.0004 | 0.984 | 0.996 |
| IgG *Blautia* | 1752.686 | 313.612 | 805.995 | 110.462 | 1 | 0.050 | 0.823 | 0.926 |
| IgG *Roseburia* | 58226.873 | 23107.902 | 2298.014 | 497.884 | 1 | 0.002 | 0.964 | 0.996 |
| IgG *Faecalibacterium* | 624.288 | 103.406 | 550.850 | 111.705 | 1 | 0.028 | 0.867 | 0.952 |
| IgG *Bacteroides* | 540.5166 | 111.790 | 1162.335 | 145.319 | 1 | 6.627 | 0.010 | 0.057 |
| IgG *Escherichia* | 317.849 | 40.804 | 202.379 | 25.462 | 1 | 0.716 | 0.397 | 0.715 |
| IgG *Prevotella* | 1135.428 | 200.205 | 1306.995 | 191.079 | 1 | 0.360 | 0.548 | 0.752 |
| IgG *Ruminnococcus* | 253.315 | 26.357 | 205.782 | 23.691 | 1 | 1.546 | 0.214 | 0.437 |
| IgG *Eubacterium* | 928.401 | 162.553 | 870.278 | 131.644 | 1 | 0.347 | 0.556 | 0.752 |
| IgM *Lactobacillus* | 139.852 | 13.854 | 271.938 | 23.699 | 1 | 8.208 | 0.004 | 0.047 |
| IgM *Bifidobacterium* | 268.372 | 30.364 | 215.814 | 17.014 | 1 | 0.203 | 0.652 | 0.816 |
| IgM *Blautia* | 467.471 | 67.410 | 719.260 | 46.926 | 1 | 4.108 | 0.043 | 0.150 |
| IgM *Roseburia* | 560.861 | 64.700 | 507.405 | 47.748 | 1 | 0.00002 | 0.996 | 0.996 |
| IgM *Faecalibacterium* | 449.116 | 58.278 | 728.557 | 89.460 | 1 | 3.279 | 0.070 | 0.211 |
| IgM *Bacteroides* | 214.526 | 22.107 | 331.268 | 24.968 | 1 | 4.928 | 0.026 | 0.119 |
| IgM *Escherichia* | 258.880 | 25.246 | 300.337 | 17.968 | 1 | 0.567 | 0.451 | 0.752 |
| IgM *Prevotella* | 674.304 | 64.687 | 738.125 | 67.207 | 1 | 0.326 | 0.568 | 0.752 |
| IgM *Ruminnococcus* | 950.882 | 105.168 | 1410.136 | 130.170 | 1 | 1.965 | 0.161 | 0.345 |
| IgM *Eubacterium* | 327.393 | 44.645 | 257.624 | 22.900 | 1 | 0.124 | 0.725 | 0.882 |
| L-FABP | 43830.471 | 4160.174 | 48551.343 | 6318.161 | 1 | 0.077 | 0.782 | 0.902 |
| TIMP-1 | 334753.742 | 10787.605 | 485399.304 | 54082.472 | 1 | 1.111 | 0.292 | 0.555 |
| I-FABP | 2283.432 | 355.621 | 2088.090 | 293.455 | 1 | 1.092 | 0.296 | 0.555 |
| MBL | 342892.528 | 16767.455 | 367682.465 | 37131.658 | 1 | 0.004 | 0.952 | 0.996 |
| OPG | 1621.658 | 78.409 | 2489.978 | 159.676 | 1 | 9.476 | 0.002 | 0.031 |
| MMP-9 | 1068074.171 | 77856.609 | 1357706.526 | 96748.331 | 1 | 2.276 | 0.131 | 0.311 |
| EG-VEGF | 37.439 | 25.530 | 147.071 | 60.440 | 1 | 6.620 | 0.010 | 0.057 |
| LBP | 8178.638 | 279.458 | 9291.492 | 725.877 | 1 | 0.080 | 0.777 | 0.902 |
| CD14 | 2072109.067 | 69294.709 | 1798876.052 | 61994.938 | 1 | 2.809 | 0.094 | 0.248 |
| TFF-3 | 8413.252 | 2096.085 | 13335.618 | 3172.523 | 1 | 5.070 | 0.024 | 0.119 |
| TGF-β1 | 17287.187 | 1253.498 | 89053.521 | 10326.956 | 1 | 17.322 | <0.001 | 0.001 |
| IGF2 | 69769.930 | 24423.061 | 1390636.981 | 495150.368 | 1 | 2.278 | 0.131 | 0.311 |
| TNF-α | 29.681 | 23.391 | 95.805 | 28.277 | 1 | 6.984 | 0.008 | 0.057 |
| IL-18 | 453.694 | 28.195 | 563.076 | 27.662 | 1 | 2.978 | 0.084 | 0.237 |
| IL-33 | 0 | 0 | 2178.636 | 913.647 | 1 | 10.528 | 0.001 | 0.026 |
