# Supplementary material for: Association between ustekinumab therapy and changes in specific anti-microbial response, serum biomarkers, and microbiota composition in patients with IBD: A pilot study
Source: PLoS One. 2022 Dec 30;17(12):e0277576. doi: 10.1371/journal.pone.0277576 (PMC9803183; doi:10.1371/journal.pone.0277576)
Supplement: S5 Table — Longitudinal changes were modeled via linear mixed effect models, where the temporal variation was fitted as a linear continuous predictor, quadratic polynomial term, piecewise polynomials term fitted via B splines or as a categorial predictor. Performance of alternative models was assessed based on Akaike information criterion difference (ΔAIC) between the best fitting model and corresponding AIC weights. P values were derived based on the deviance change between the null model and the best fitting non-null model assuming its χ2 distribution. Q value method for estimating false discovery rate was used to correct for multiple hypothesis testing. (DOCX) [file pone.0277576.s007.docx]

**Supplementary Table 5**: Temporal variation of serum biomarker concentrations in patients with IBD treated with ustekinumab. Longitudinal changes were modeled via linear mixed effect models, where the temporal variation was fitted as a linear continuous predictor, quadratic polynomial term, piecewise polynomials term fitted via B splines or as a categorial predictor. Performance of alternative models was assessed based on Akaike information criterion difference (ΔAIC) between the best fitting model and corresponding AIC weights. *P* values were derived based on the deviance change between the null model and the best fitting non-null model assuming its χ^2^ distribution. *Q* value method for estimating false discovery rate was used to correct for multiple hypothesis testing.

| **Model** | **Categorial** | | **Quadratic** | | **B-splines** | | **Linear** | | **Null** | |  |  |
| --- | --- | --- | --- | --- | --- | --- | --- | --- | --- | --- | --- | --- |
| **Biomarker** | **ΔAIC** | **AIC weights** | **ΔAIC** | **AIC weights** | **ΔAIC** | **AIC weights** | **ΔAIC** | **AIC weights** | **ΔAIC** | **AIC weights** | ***p* value** | ***q* value** |
| IgA *Lactobacillus* | 7.844 | 0.012 | 3.932 | 0.088 | 5.907 | 0.033 | 1.942 | 0.238 | 0.000 | 0.629 | 0.809 | 0.398 |
| IgA *Bifidobacterium* | 2.840 | 0.079 | 0.176 | 0.300 | 1.982 | 0.122 | 1.299 | 0.171 | 0.000 | 0.328 | 0.148 | 0.161 |
| IgA *Blautia* | 4.657 | 0.051 | 3.662 | 0.084 | 2.717 | 0.134 | 1.847 | 0.208 | 0.000 | 0.523 | 0.696 | 0.378 |
| IgA *Roseburia* | 5.184 | 0.038 | 1.985 | 0.187 | 3.437 | 0.091 | 0.000 | 0.506 | 2.088 | 0.178 | 0.043 | 0.127 |
| IgA *Faecalibacterium* | 1.141 | 0.151 | 0.082 | 0.257 | 0.706 | 0.188 | 0.000 | 0.267 | 1.333 | 0.137 | 0.068 | 0.127 |
| IgA *Bacteroides* | 6.220 | 0.026 | 3.538 | 0.100 | 4.360 | 0.066 | 1.960 | 0.220 | 0.000 | 0.587 | 0.841 | 0.402 |
| IgA *Escherichia* | 7.623 | 0.014 | 3.750 | 0.095 | 5.633 | 0.037 | 1.970 | 0.232 | 0.000 | 0.622 | 0.863 | 0.403 |
| IgA *Prevotella* | 6.595 | 0.018 | 2.771 | 0.125 | 4.767 | 0.046 | 0.929 | 0.313 | 0.000 | 0.498 | 0.301 | 0.237 |
| IgA *Ruminnococcus* | 6.754 | 0.021 | 3.725 | 0.094 | 5.130 | 0.047 | 1.944 | 0.230 | 0.000 | 0.608 | 0.813 | 0.398 |
| IgA *Eubacterium* | 2.493 | 0.110 | 3.017 | 0.085 | 1.098 | 0.221 | 1.277 | 0.202 | 0.000 | 0.382 | 0.179 | 0.184 |
| IgG *Lactobacillus* | 4.505 | 0.042 | 1.690 | 0.172 | 3.646 | 0.065 | 0.435 | 0.322 | 0.000 | 0.400 | 0.211 | 0.196 |
| IgG *Bifidobacterium* | 1.997 | 0.136 | 2.239 | 0.120 | 0.000 | 0.369 | 0.374 | 0.306 | 3.371 | 0.068 | 0.025 | 0.087 |
| IgG *Blautia* | 2.759 | 0.097 | 0.983 | 0.235 | 1.328 | 0.198 | 0.000 | 0.384 | 2.997 | 0.086 | 0.025 | 0.087 |
| IgG *Roseburia* | 3.625 | 0.082 | 1.827 | 0.201 | 2.578 | 0.138 | 0.000 | 0.502 | 3.747 | 0.077 | 0.017 | 0.087 |
| IgG *Faecalibacterium* | 3.639 | 0.075 | 0.964 | 0.285 | 2.578 | 0.127 | 0.000 | 0.462 | 4.437 | 0.050 | 0.011 | 0.087 |
| IgG *Bacteroides* | 3.757 | 0.058 | 2.352 | 0.116 | 1.820 | 0.152 | 0.468 | 0.298 | 0.000 | 0.377 | 0.216 | 0.196 |
| IgG *Escherichia* | 2.686 | 0.111 | 1.788 | 0.174 | 1.422 | 0.209 | 0.000 | 0.426 | 3.367 | 0.079 | 0.021 | 0.087 |
| IgG *Prevotella* | 4.183 | 0.056 | 2.555 | 0.126 | 3.993 | 0.062 | 0.811 | 0.302 | 0.000 | 0.454 | 0.275 | 0.236 |
| IgG *Ruminnococcus* | 1.971 | 0.178 | 3.184 | 0.097 | 5.020 | 0.039 | 1.640 | 0.210 | 0.000 | 0.476 | 0.548 | 0.331 |
| IgG *Eubacterium* | 0.323 | 0.286 | 2.280 | 0.108 | 3.545 | 0.057 | 0.916 | 0.213 | 0.000 | 0.336 | 0.104 | 0.153 |
| IgM *Lactobacillus* | 7,164 | 0,017 | 3,562 | 0,102 | 5,192 | 0,045 | 1,934 | 0,230 | 0,000 | 0,606 | 0,797 | 0,398 |
| IgM *Bifidobacterium* | 7,549 | 0,014 | 3,792 | 0,091 | 5,549 | 0,038 | 1,792 | 0,248 | 0,000 | 0,608 | 0,649 | 0,378 |
| IgM *Blautia* | 2,135 | 0,131 | 2,893 | 0,090 | 1,395 | 0,190 | 1,205 | 0,209 | 0,000 | 0,381 | 0,373 | 0,255 |
| IgM *Roseburia* | 0,000 | 0,304 | 2,040 | 0,110 | 0,395 | 0,250 | 0,084 | 0,292 | 3,806 | 0,045 | 0,019 | 0,087 |
| IgM *Faecalibacterium* | 5,834 | 0,028 | 2,066 | 0,184 | 3,996 | 0,070 | 1,869 | 0,203 | 0,000 | 0,516 | 0,718 | 0,378 |
| IgM *Bacteroides* | 7,063 | 0,016 | 3,337 | 0,105 | 5,147 | 0,042 | 1,357 | 0,282 | 0,000 | 0,555 | 0,423 | 0,280 |
| IgM *Escherichia* | 1,906 | 0,113 | 1,931 | 0,111 | 0,362 | 0,244 | 0,395 | 0,240 | 0,000 | 0,292 | 0,131 | 0,161 |
| IgM *Prevotella* | 5,301 | 0,030 | 1,531 | 0,198 | 3,378 | 0,079 | 0,000 | 0,426 | 0,926 | 0,268 | 0,087 | 0,149 |
| IgM *Ruminnococcus* | 0,000 | 0,296 | 0,791 | 0,199 | 0,833 | 0,195 | 1,771 | 0,122 | 0,898 | 0,189 | 0,064 | 0,127 |
| IgM *Eubacterium* | 5,340 | 0,029 | 1,980 | 0,154 | 3,502 | 0,072 | 0,000 | 0,413 | 0,432 | 0,333 | 0,119 | 0,161 |
| L-FABP | 4,495 | 0,051 | 1,725 | 0,205 | 3,713 | 0,076 | 1,948 | 0,183 | 0,000 | 0,485 | 0,321 | 0,237 |
| TIMP-1 | 4,862 | 0,045 | 3,453 | 0,092 | 3,267 | 0,100 | 1,455 | 0,248 | 0,000 | 0,514 | 0,461 | 0,296 |
| I-FABP | 5,408 | 0,034 | 2,792 | 0,124 | 4,719 | 0,047 | 1,060 | 0,295 | 0,000 | 0,501 | 0,332 | 0,237 |
| MBL | 7,098 | 0,017 | 3,268 | 0,116 | 5,267 | 0,043 | 1,867 | 0,233 | 0,000 | 0,592 | 0,715 | 0,378 |
| OPG | 4,408 | 0,056 | 2,484 | 0,147 | 3,243 | 0,100 | 1,984 | 0,189 | 0,000 | 0,508 | 0,898 | 0,410 |
| MMP-9 | 3,232 | 0,068 | 0,000 | 0,343 | 1,860 | 0,135 | 1,188 | 0,190 | 0,529 | 0,264 | 0,104 | 0,153 |
| EG-VEGF | 2,469 | 0,095 | 2,652 | 0,087 | 0,486 | 0,257 | 0,678 | 0,233 | 0,000 | 0,327 | 0,138 | 0,161 |
| LBP | 1,477 | 0,166 | 0,966 | 0,214 | 2,062 | 0,124 | 0,000 | 0,348 | 1,709 | 0,148 | 0,054 | 0,127 |
| CD14 | 0,919 | 0,212 | 0,963 | 0,207 | 0,000 | 0,336 | 3,194 | 0,068 | 1,282 | 0,177 | 0,063 | 0,127 |
| TFF-3 | 4,010 | 0,068 | 3,274 | 0,099 | 3,418 | 0,092 | 1,536 | 0,235 | 0,000 | 0,507 | 0,496 | 0,309 |
| TGF-β1 | 4,614 | 0,038 | 1,247 | 0,203 | 2,967 | 0,086 | 0,491 | 0,296 | 0,000 | 0,378 | 0,219 | 0,196 |
| IGF2 | 6,294 | 0,022 | 2,935 | 0,118 | 4,708 | 0,049 | 1,067 | 0,300 | 0,000 | 0,511 | 0,334 | 0,237 |
| TNF-α | 4,017 | 0,072 | 3,151 | 0,110 | 4,027 | 0,071 | 1,837 | 0,213 | 0,000 | 0,534 | 0,687 | 0,378 |
| IL-18 | 6,222 | 0,023 | 2,987 | 0,114 | 4,812 | 0,046 | 0,988 | 0,310 | 0,000 | 0,508 | 0,314 | 0,237 |
| IL-33 | 3,899 | 0,052 | 0,191 | 0,330 | 2,191 | 0,121 | 1,999 | 0,134 | 0,000 | 0,363 | 0,149 | 0,161 |
