# Supplementary material for: Association between ustekinumab therapy and changes in specific anti-microbial response, serum biomarkers, and microbiota composition in patients with IBD: A pilot study
Source: PLoS One. 2022 Dec 30;17(12):e0277576. doi: 10.1371/journal.pone.0277576 (PMC9803183; doi:10.1371/journal.pone.0277576)
Supplement: S6 Table — Mean values and their standard deviations (in parentheses) are shown. P values were derived on the basis of the likelihood ratio test, which assumes a χ2 distribution of deviance changes. The Q-value method for estimating the false discovery rate was used to correct for multiple hypothesis testing. (DOCX) [file pone.0277576.s008.docx]

**Supplementary Table 6**: Change in serum biomarker concentrations in patients with IBD treated with ustekinumab between week 0 and week 40. Mean values and their standard deviations (in parentheses) corresponding to arbitrary units (AU) in the case of antibodies against bacteria and pg/ml in the case of other biomarkers are shown. P values were derived on the basis of the likelihood ratio test, which assumes a χ2 distribution of deviance changes between models estimating the difference between the two time points and their simplified version estimating just the total intercept. The Q-value method for estimating the false discovery rate was used to correct for multiple hypothesis testing.

| **Biomarker** | **Week 0** | **Week 40** | **ΔD.F.** | **χ^2^** | **p** | **q** |
| --- | --- | --- | --- | --- | --- | --- |
| *Lactobacillus*_IgA | 479.751 (266.524) | 414.817 (180.832) | 1 | 0.1339 | 0.7144 | 0.5158 |
| *Bifidobacterium*_IgA | 481.928 (224.162) | 532.541 (288.246) | 1 | 0.7122 | 0.3987 | 0.4552 |
| *Blautia*_IgA | 678.423 (552.43) | 1239.429 (1563.364) | 1 | 1.3624 | 0.2431 | 0.4505 |
| *Roseburia*_IgA | 720.043 (1183.937) | 804.713 (1322.882) | 1 | 5.1358 | 0.0234 | 0.4099 |
| *Faecalibacterium*_IgA | 242.21 (255.97) | 317.304 (321.95) | 1 | 2.0646 | 0.1508 | 0.4505 |
| *Bacteroides*_IgA | 322.134 (213.707) | 303.207 (210.4) | 1 | 0.1496 | 0.6989 | 0.5158 |
| *Escherichia*_IgA | 220.673 (102.032) | 220.355 (135.833) | 1 | 0.1715 | 0.6788 | 0.5158 |
| *Prevotella*_IgA | 1041.891 (852.368) | 901.414 (673.799) | 1 | 0.6034 | 0.4373 | 0.4792 |
| *Ruminnococcus*_IgA | 471.156 (234.085) | 487.583 (330.607) | 1 | 0.1998 | 0.6549 | 0.5158 |
| *Eubacterium*_IgA | 703.1 (438.658) | 804.234 (892.841) | 1 | 0.9801 | 0.3222 | 0.4505 |
| *Lactobacillus*_IgG | 447.654 (292.014) | 536.911 (476.491) | 1 | 1.6133 | 0.2040 | 0.4505 |
| *Bifidobacterium*_IgG | 842.174 (1054.297) | 1030.638 (1439.507) | 1 | 0.3955 | 0.5294 | 0.5158 |
| *Blautia*_IgG | 670.003 (471.78) | 836.711 (704.968) | 1 | 1.9734 | 0.1601 | 0.4505 |
| *Roseburia*_IgG | 2132.945 (3294.41) | 2832.351 (4518.377) | 1 | 1.9897 | 0.1584 | 0.4505 |
| *Faecalibacterium*_IgG | 505.139 (656.096) | 719.899 (1154.254) | 1 | 2.5040 | 0.1136 | 0.4505 |
| *Bacteroides*_IgG | 1108.299 (693.237) | 1141.844 (969.432) | 1 | 0.1329 | 0.7154 | 0.5158 |
| *Escherichia*_IgG | 174.854 (127.095) | 239.023 (215.978) | 1 | 1.3161 | 0.2513 | 0.4505 |
| *Prevotella*_IgG | 1300.122 (984.441) | 1163.906 (1101.4) | 1 | 1.2095 | 0.2714 | 0.4505 |
| *Ruminnococcus*_IgG | 203.378 (154.505) | 205.106 (152.064) | 1 | 0.1478 | 0.7007 | 0.5158 |
| *Eubacterium*_IgG | 952.984 (1057.467) | 864.142 (828.947) | 1 | 0.3104 | 0.5774 | 0.5158 |
| *Lactobacillus*_IgM | 277.475 (175.513) | 272.088 (130.831) | 1 | 0.0051 | 0.9429 | 0.5741 |
| *Bifidobacterium*_IgM | 208.757 (135.773) | 221.057 (114.575) | 1 | 0.9024 | 0.3421 | 0.4505 |
| *Blautia*_IgM | 703.049 (269.558) | 709.341 (266.323) | 1 | 0.0429 | 0.8360 | 0.5586 |
| *Roseburia*_IgM | 469.558 (273.147) | 514.426 (341.856) | 1 | 0.8810 | 0.3479 | 0.4505 |
| *Faecalibacterium*_IgM | 742.57 (592.919) | 780.61 (801.936) | 1 | 0.0777 | 0.7804 | 0.5346 |
| *Bacteroides*_IgM | 330.665 (166.352) | 327.946 (154.616) | 1 | 0.2710 | 0.6027 | 0.5158 |
| *Escherichia*_IgM | 293.676 (125.357) | 315.107 (117.656) | 1 | 0.0322 | 0.8575 | 0.5594 |
| *Prevotella*_IgM | 728.919 (423.598) | 721.998 (440.643) | 1 | 1.6006 | 0.2058 | 0.4505 |
| *Ruminnococcus*_IgM | 1325.33 (775.932) | 1317.849 (833.661) | 1 | 0.0902 | 0.7639 | 0.5346 |
| *Eubacterium*_IgM | 259.242 (168.01) | 246.696 (114.959) | 1 | 0.7705 | 0.3801 | 0.4527 |
| L_FABP | 46881.583 (45862.934) | 43933.761 (42621.354) | 1 | 0.5339 | 0.4650 | 0.4900 |
| TIMP_1 | 532403.703 (309888.979) | 403588.117 (231517.451) | 1 | 2.8110 | 0.0936 | 0.4505 |
| I_FABP | 1651.2 (1382.361) | 2266.785 (2526.993) | 1 | 0.8541 | 0.3554 | 0.4505 |
| MBL | 372501.711 (216042.982) | 373649.831 (269241.407) | 1 | 0.0076 | 0.9306 | 0.5741 |
| osteo | 2364.331 (1073.477) | 2451.37 (887.24) | 1 | 0.1684 | 0.6815 | 0.5158 |
| MMP_9 | 1828756.606 (973304.063) | 1390572.523 (275694.912) | 1 | 1.3966 | 0.2373 | 0.4505 |
| EG_VEGF | 122.563 (310.467) | 225.592 (552.561) | 1 | 3.0611 | 0.0802 | 0.4505 |
| LBP | 9765.236 (3885.946) | 7942.567 (5205.236) | 1 | 4.7139 | 0.0299 | 0.4099 |
| CD_14 | 1784805.975 (414711.801) | 1668831.201 (417180.858) | 1 | 0.8318 | 0.3617 | 0.4505 |
| TFF_3 | 15965.905 (26515.035) | 10153.452 (9101.921) | 1 | 0.0108 | 0.9173 | 0.5741 |
| TGF_ β 1 | 82757.747 (70977.36) | 92091.854 (62791.612) | 1 | 0.2722 | 0.6019 | 0.5158 |
| IGF_II | 553921.23 (1143143.91) | 1839290.45 (4766232.121) | 1 | 0.1957 | 0.6582 | 0.5158 |
| TNF_alpha | 55.343 (118.08) | 77.937 (220.41) | 1 | 1.5430 | 0.2142 | 0.4505 |
| IL_18 | 573.923 (186.089) | 543.637 (205.82) | 1 | 1.0313 | 0.3098 | 0.4505 |
| IL_33 | 1601.623 (4447.666) | 2668.718 (7548.247) | 1 | 1.0679 | 0.3014 | 0.4505 |
