# Supplementary material for: Association between ustekinumab therapy and changes in specific anti-microbial response, serum biomarkers, and microbiota composition in patients with IBD: A pilot study
Source: PLoS One. 2022 Dec 30;17(12):e0277576. doi: 10.1371/journal.pone.0277576 (PMC9803183; doi:10.1371/journal.pone.0277576)
Supplement: S7 Table — Values for test statistics (Χ2), associated degrees of freedom (DF) and resulting p values are shown. ASV (amplicon sequence variants). (DOCX) [file pone.0277576.s009.docx]

**Supplementary Table 7:** Results of linear mixed effect models testing for differences in alpha diversity metrics between the stool microbiome of patients with IBD and healthy controls. Values for test statistics (Χ^2^), associated degrees of freedom (DF) and resulting *p* values are shown. ASV (amplicon sequence variants).

| **Community** | **Alpha diversity metric** | **DF** | **Χ^2^** | ***p* value** | ***q* value** |
| --- | --- | --- | --- | --- | --- |
| Bacteriome | **Observed ASVs** | 1 | 1.208 | 0.272 | 0.978 |
|  | **Chao1** | 1 | 1.255 | 0.263 | 0.978 |
|  | **Faith's phylogenetic diversity** | 1 | 17.055 | < 0.001 | 0.0003 |
|  | **Shannon entropy** | 1 | 0.226 | 0.634 | 0.978 |
| Mycobiome | **Observed ASVs** | 1 | 0.001 | 0.978 | 0.978 |
|  | **Chao1** | 1 | 0.098 | 0.754 | 0.978 |
|  | **Shannon entropy** | 1 | 1.100 | 0.294 | 0.978 |
