# Supplementary material for: Association between ustekinumab therapy and changes in specific anti-microbial response, serum biomarkers, and microbiota composition in patients with IBD: A pilot study
Source: PLoS One. 2022 Dec 30;17(12):e0277576. doi: 10.1371/journal.pone.0277576 (PMC9803183; doi:10.1371/journal.pone.0277576)
Supplement: S8 Table — Values for test statistics (H), associated degrees of freedom (DF) and resulting p and q values are shown. ASV (amplicon sequence variants), HC (healthy controls), IBD (inflammatory bowel disease). (DOCX) [file pone.0277576.s010.docx]

**Supplementary Table 8:** Results of non-parametric Kruskal-Wallis test comparing differences in alpha diversity metrics between the stool microbiome of patients with IBD and healthy controls **A)** at baseline (week 0) and **B)** endpoint (week 40). Values for test statistics (H), associated degrees of freedom (DF) and resulting *p* and *q* values are shown. ASV (amplicon sequence variants), HC (healthy controls), IBD (inflammatory bowel disease).

|  |  | **A) IBD vs HC week 0** | | | | **B) IBD vs HC week 40** | | | |
| --- | --- | --- | --- | --- | --- | --- | --- | --- | --- |
| **Community** | **Alpha diversity metric** | **DF** | **H** | ***p* value** | ***q* value** | **DF** | **H** | ***p* value** | ***q* value** |
| Bacteriome | **Observed ASVs** | 1 | 0.096168566 | 0.75647687 | 1.000000000 | 1 | 0.405719411 | 0.524150199 | 1.00000000 |
|  | **Chao1** | 1 | 0.104010613 | 0.74706881 | 1.000000000 | 1 | 0.405719411 | 0.524150199 | 1.00000000 |
|  | **Faith's phylogenetic diversity** | 1 | 14.21784615 | 0.00016282 | 0.000651276 | 1 | 2.603365385 | 0.106637058 | 0.42654823 |
|  | **Shannon entropy** | 1 | 2.442461538 | 0.11809078 | 0.472363139 | 1 | 0.007211538 | 0.932324373 | 1.00000000 |
| Mycobiome | **Observed ASVs** | 1 | 0.756639131 | 0.38438235 | 1.000000000 | 1 | 0.03840859 | 0.844624962 | 1.00000000 |
|  | **Chao1** | 1 | 0.012863039 | 0.90970128 | 1.000000000 | 1 | 0.835533553 | 0.360677309 | 1.00000000 |
|  | **Shannon entropy** | 1 | 0.412857143 | 0.52052288 | 1.000000000 | 1 | 0.017045455 | 0.896124778 | 1.00000000 |
