# Supplementary material for: Association between ustekinumab therapy and changes in specific anti-microbial response, serum biomarkers, and microbiota composition in patients with IBD: A pilot study
Source: PLoS One. 2022 Dec 30;17(12):e0277576. doi: 10.1371/journal.pone.0277576 (PMC9803183; doi:10.1371/journal.pone.0277576)
Supplement: S9 Table — Longitudinal changes were modeled via linear mixed effect models, where the temporal variation was fitted as linear continuous predictor, quadratic polynomial term, piecewise polynomials term fitted via B splines or as a categorial predictor. Performance of alternative models was assessed based on Akaike information criterion difference (ΔAIC) between the best fitting model and corresponding AIC weights. P values were derived based on the deviance change between the null model and the best fitting non-null model assuming its χ2 distribution. Q value method for estimating false discovery rate was used to correct for multiple hypothesis testing. PD (phylogenetic diversity), ASV (amplicon sequence variants). (DOCX) [file pone.0277576.s011.docx]

**Supplementary Table 9:** Temporal variation of stool microbial alpha diversity of patients with IBD treated with ustekinumab. Longitudinal changes were modeled via linear mixed effect models, where the temporal variation was fitted as linear continuous predictor, quadratic polynomial term, piecewise polynomials term fitted via B splines or as a categorial predictor. Performance of alternative models was assessed based on Akaike information criterion difference (ΔAIC) between the best fitting model and corresponding AIC weights. *P* values were derived based on the deviance change between the null model and the best fitting non-null model assuming its χ^2^ distribution. *Q* value method for estimating false discovery rate was used to correct for multiple hypothesis testing. PD (phylogenetic diversity), ASV (amplicon sequence variants).

|  | | **Model** | **Categorical** | | **Quadratic** | | **B-splines** | | **Linear** | | **Null** | |  |  |
| --- | --- | --- | --- | --- | --- | --- | --- | --- | --- | --- | --- | --- | --- | --- |
|  | **Alpha diversity metric** | | **ΔAIC** | **weights AIC** | **ΔAIC** | **weights AIC** | **ΔAIC** | **weights AIC** | **ΔAIC** | **weights AIC** | **ΔAIC** | **weights AIC** | ***p* value** | ***q* value** |
| Bacteriome | **Faith's PD** | | 5.027 | 0.039 | 3.103 | 0.103 | 1.876 | 0.191 | 1.988 | 0.180 | 0.000 | 0.487 | 0.248 | 0.124 |
|  | **Chao1** | | 5.874 | 0.023 | 2.057 | 0.156 | 2.441 | 0.128 | 1.046 | 0.258 | 0.000 | 0.435 | 0.329 | 0.124 |
|  | **observed ASVs** | | 6.096 | 0.021 | 2.042 | 0.157 | 2.622 | 0.117 | 0.951 | 0.270 | 0.000 | 0.435 | 0.306 | 0.124 |
|  | **Shannon entropy** | | 3.157 | 0.068 | 1.243 | 0.178 | 1.177 | 0.184 | 0.000 | 0.331 | 0.639 | 0.240 | 0.104 | 0.124 |
| Mycobiome | **Chao1** | | 8.012 | 0.009 | 2.591 | 0.129 | 4.576 | 0.048 | 0.636 | 0.343 | 0.000 | 0.471 | 0.243 | 0.124 |
|  | **observed ASVs** | | 6.657 | 0.016 | 1.910 | 0.170 | 3.239 | 0.088 | 0.000 | 0.443 | 0.889 | 0.284 | 0.089 | 0.124 |
|  | **Shannon entropy** | | 6.479 | 0.015 | 1.417 | 0.191 | 2.638 | 0.104 | 0.509 | 0.301 | 0.000 | 0.388 | 0.222 | 0.124 |
