# Supplementary material for: Association between ustekinumab therapy and changes in specific anti-microbial response, serum biomarkers, and microbiota composition in patients with IBD: A pilot study
Source: PLoS One. 2022 Dec 30;17(12):e0277576. doi: 10.1371/journal.pone.0277576 (PMC9803183; doi:10.1371/journal.pone.0277576)
Supplement: S10 Table — Values for test statistics (H), associated degrees of freedom (DF) and resulting p and q values are shown. ASV (amplicon sequence variants), CD (Crohn’s disease), UC (ulcerative colitis). (DOCX) [file pone.0277576.s012.docx]

**Supplementary Table 10:** Results of non-parametric Kruskal-Wallis test comparing differences in alpha diversity metrics between the stool microbiome of patients with CD and UC **A)** at baseline (week 0) and **B)** endpoint (week 40). Values for test statistics (H), associated degrees of freedom (DF) and resulting *p* and *q* values are shown. ASV (amplicon sequence variants), CD (Crohn’s disease), UC (ulcerative colitis)

|  |  | **A) CD vs UC week 0** | | | | **B) CD vs UC week 40** | | | |
| --- | --- | --- | --- | --- | --- | --- | --- | --- | --- |
| **Community** | **Alpha diversity metric** | **DF** | **H** | ***p* value** | ***q* value** | **DF** | **H** | ***p* value** | ***q* value** |
| Bacteriome | **Observed ASVs** | 1 | 2.454545455 | 0.117185087 | 0.468740350 | 1 | 0.111111111 | 0.738882680 | 0.182001060 |
|  | **Chao1** | 1 | 2.454545455 | 0.117185087 | 0.468740350 | 1 | 0.111111111 | 0.738882680 | 0.182001060 |
|  | **Faith's phylogenetic diversity** | 1 | 0.068181818 | 0.794002680 | 1.000000000 | 1 | 0.444444444 | 0.504985075 | 0.729689760 |
|  | **Shannon entropy** | 1 | 1.090909091 | 0.296269871 | 1.000000000 | 1 | 0.111111111 | 0.738882680 | 0.382322820 |
| Mycobiome | **Observed ASVs** | 1 | 0.017149390 | 0.895810367 | 1.000000000 | 1 | 0.111111111 | 0.738882680 | 1.000000000 |
|  | **Chao1** | 1 | 0.274390244 | 0.600401848 | 1.000000000 | 1 | 0.111111111 | 0.738882680 | 1.000000000 |
|  | **Shannon entropy** | 1 | 0.000000000 | 1.000000000 | 1.000000000 | 1 | 2.777777778 | 0.095580705 | 1.000000000 |
