# Supplementary material for: Association between ustekinumab therapy and changes in specific anti-microbial response, serum biomarkers, and microbiota composition in patients with IBD: A pilot study
Source: PLoS One. 2022 Dec 30;17(12):e0277576. doi: 10.1371/journal.pone.0277576 (PMC9803183; doi:10.1371/journal.pone.0277576)
Supplement: S11 Table — MDMR testing differences in community composition A) between IBD patients and healthy controls. The week of sample collection was fitted as a B) categorial or C) continuous predictor. Values for test statistics, associated degrees of freedom (DF) and resulting p values are shown. IBD (patients with inflammatory bowel disease), HC (healthy controls), w (weighted), u (unweighted). (DOCX) [file pone.0277576.s013.docx]

**Supplementary Table 11:** Results of multivariate distance matrix regression (MDMR) testing for variation in stool microbiome composition of patients with IBD during ustekinumab treatment. MDMR testing differences in community composition **A)** between IBD patients and healthy controls. The week of sample collection was fitted as a **B)** categorial or **C)** continuous predictor. Values for test statistics, associated degrees of freedom (DF) and resulting *p* values are shown. IBD (patients with inflammatory bowel disease), HC (healthy controls), w (weighted), u (unweighted).

|  |  | **A) IBD vs HC** | | | | **B) week categorical** | | | | **C) week continuous** | | | |
| --- | --- | --- | --- | --- | --- | --- | --- | --- | --- | --- | --- | --- | --- |
| **Community** | **Distance metric** | **DF** | **Test statistic** | ***p***  **value** | ***q***  **value** | **DF** | **Test statistic** | ***p* value** | ***q***  **value** | **DF** | **Test statistic** | ***p* value** | ***q***  **value** |
| Bacteriome | Bray-Curtis | 1 | 3.612 | 1.13E-08 | 4.51E-08 | 5 | 4.822 | 0.575 | 0.630 | 1 | 1.032 | 0.399 | 0.544 |
|  | Jaccard | 1 | 3.014 | 8.91E-09 | 4.46E-08 | 5 | 5.763 | 0.094 | 0.566 | 1 | 1.591 | 0.023 | 0.140 |
|  | w. UniFrac | 1 | 8.330 | 1.29E-06 | 3.87E-06 | 5 | 4.883 | 0.479 | 0.630 | 1 | 1.697 | 0.118 | 0.544 |
|  | u. UniFrac | 1 | 11.662 | 2.23E-14 | 1.34E-13 | 5 | 5.828 | 0.137 | 0.630 | 1 | 0.980 | 0.459 | 0.544 |
| Mycobiome | Bray-Curtis | 1 | 2.941 | 1.13E-03 | 1.13E-03 | 5 | 4.536 | 0.630 | 0.630 | 1 | 0.852 | 0.544 | 0.544 |
|  | Jaccard | 1 | 2.047 | 1.76E-04 | 3.51E-04 | 5 | 5.394 | 0.216 | 0.630 | 1 | 0.980 | 0.491 | 0.544 |
