# Supplementary material for: Association between ustekinumab therapy and changes in specific anti-microbial response, serum biomarkers, and microbiota composition in patients with IBD: A pilot study
Source: PLoS One. 2022 Dec 30;17(12):e0277576. doi: 10.1371/journal.pone.0277576 (PMC9803183; doi:10.1371/journal.pone.0277576)
Supplement: S12 Table — Microbial beta diversity variability between a group of healthy controls (HC) and patients with IBD A) at the baseline (week 0), B) at the endpoint (week 40) was assessed with permutational tests PERMANOVA and PERMDISP using 1000 permutations. Values for test statistics, p values, adjusted q values and degrees of freedom (DF) are reported. (DOCX) [file pone.0277576.s014.docx]

**Supplementary Table 12:** Microbial beta diversity variability between a group of healthy controls (HC) and patients with IBD **A**) at the baseline (week 0), **B**) at the endpoint (week 40) was assessed with permutational tests PERMANOVA and PERMDISP using 1000 permutations. Values for test statistics, *p* values, adjusted *q* values and degrees of freedom (DF) are reported.

|  |  | **A) IBD vs HC week 0** | | | | | |  |
| --- | --- | --- | --- | --- | --- | --- | --- | --- |
|  |  |  | **PERMANOVA** | |  | **PERMDISP** | |  |
| **Community** | **Beta diversity metric** | **DF** | **pseudo-F** | ***p* value** | ***q value*** | **F-value** | ***p* value** | ***q* value** |
| Bacteriome | **Bray-Curtis** | 1 | 2.19279 | 0.00100 | 0.00400 | 0.117196 | 0.95700 | 1.000 |
|  | **Jaccard** | 1 | 1.94099 | 0.00100 | 0.00400 | 4.225900 | 0.85500 | 1.000 |
|  | **unweighted UniFrac** | 1 | 5.73198 | 0.00100 | 0.00400 | 4.841430 | 0.07200 | 0.288 |
|  | **weighted UniFrac** | 1 | 5.20475 | 0.00100 | 0.00400 | 14.150200 | 0.00100 | 0.004 |
| Mycobiome | **Bray-Curtis** | 1 | 2.17267 | 0.00300 | 0.00600 | 5.839290 | 0.03100 | 0.062 |
|  | **Jaccard** | 1 | 1.58094 | 0.00100 | 0.00200 | 6.944820 | 0.27600 | 0.552 |
|  |  |  |  |  |  |  |  |  |
|  |  |  |  |  |  |  |  |  |
|  |  | **B) IBD vs HC week 40** | | | | | |  |
|  |  |  | **PERMANOVA** | |  | **PERMDISP** | |  |
| **Community** | **Beta diversity metric** | **DF** | **pseudo-F** | ***p* value** | ***q* value** | **F-value** | ***p* value** | ***q* value** |
| Bacteriome | **Bray-Curtis** | 1 | 2.03467 | 0.00100 | 0.00400 | 2.50005 | 0.86800 | 1.000 |
|  | **Jaccard** | 1 | 1.83778 | 0.00100 | 0.00400 | 20.94960 | 0.36300 | 1.000 |
|  | **unweighted UniFrac** | 1 | 4.40350 | 0.00100 | 0.00400 | 0.47721 | 0.60800 | 1.000 |
|  | **weighted UniFrac** | 1 | 7.01851 | 0.00100 | 0.00400 | 2.00334 | 0.19800 | 0.792 |
| Mycobiome | **Bray-Curtis** | 1 | 1.13408 | 0.24700 | 0.49400 | 2.74322 | 0.28400 | 0.568 |
|  | **Jaccard** | 1 | 1.57381 | 0.00100 | 0.00200 | 17.14530 | 0.261000 | 0.522 |
