# Supplementary material for: Association between ustekinumab therapy and changes in specific anti-microbial response, serum biomarkers, and microbiota composition in patients with IBD: A pilot study
Source: PLoS One. 2022 Dec 30;17(12):e0277576. doi: 10.1371/journal.pone.0277576 (PMC9803183; doi:10.1371/journal.pone.0277576)
Supplement: S14 Table — To compare individual variation between IBD patients and healthy controls, distances to group-specific centroids were calculated using the vegan::betadisper() function in R and included as the response in a mixed model with group identity as a predictor and individual identity as random intercepts. Separate models were fitted for different types of dissimilarity matrices describing beta diversity in bacterial and fungal profiles. Significance was assessed based on likelihood ratio tests assuming a χ2-distribution of deviance changes, followed by the q-value multiple testing correction method. (DOCX) [file pone.0277576.s016.docx]

**Supplementary Table 14:** Variability within the group of healthy controls and patients with IBD. To compare individual variation between IBD patients and healthy controls, distances to group-specific centroids were calculated using the vegan:betadisper() function in R and included as the response in a mixed model with group identity as a predictor and individual identity as random intercepts. Separate models were fitted for different types of dissimilarity matrices describing beta diversity in bacterial and fungal profiles. Significance was assessed based on likelihood ratio tests assuming a χ^2^-distribution of deviance changes, followed by the *q*-value multiple testing correction method.

| **Community** | **Distance** | **DF** | **Χ^2^** | ***p* value** | ***q* value** |
| --- | --- | --- | --- | --- | --- |
| Bacteriome | Bray-Curtis | 1 | 1.949 | 0.163 | 0.651 |
|  | Jaccard | 1 | 0.004 | 0.950 | 1.000 |
|  | weighted UniFrac | 1 | 16.998 | <0.001 | <0.001 |
|  | unweighted UniFrac | 1 | 1.042 | 0.307 | 0.922 |
| Microbiome | Bray-Curtis | 1 | 4.415 | 0.036 | 0.178 |
|  | Jaccard | 1 | 0.004 | 0.950 | 1.000 |
