# Supplementary material for: Association between ustekinumab therapy and changes in specific anti-microbial response, serum biomarkers, and microbiota composition in patients with IBD: A pilot study
Source: PLoS One. 2022 Dec 30;17(12):e0277576. doi: 10.1371/journal.pone.0277576 (PMC9803183; doi:10.1371/journal.pone.0277576)
Supplement: S15 Table — The table shows differentially abundant taxa determined by ANCOM2.1 between A) patients with inflammatory bowel disease (IBD) and healthy controls (HC) and between patients at B) baseline (week 0) and at the endpoint (week 40) of the study. W-statistics and centred log ratios (CLR) are shown. Wmax was 117 and 32 for the whole cohort including HC and IBD patients bacteriome and mycobiome, respectively (A). Wmax was 83 and 16 for IBD patients’ bacteriome and mycobiome, respectively (B). A 0.8W cut-off was chosen. uncultured (unctl.), structural zero (str. zero). (DOCX) [file pone.0277576.s017.docx]

**Supplementary Table 15:** Differentially abundant taxa from stool of patients with IBD and healthy controls. The table shows differentially abundant taxa determined by ANCOM2.1 between **A)** patients with inflammatory bowel disease (IBD) and healthy controls (HC) and between patients at **B)** baseline (week 0) and at the endpoint (week 40) of the study. W-statistics and centred log ratios (CLR) are shown. Wmax was 117 and 32 for the whole cohort including HC and IBD patients bacteriome and mycobiome, respectively (A). Wmax was 83 and 16 for IBD patients’ bacteriome and mycobiome, respectively (B). A 0.8W cut-off was chosen. uncultured (unctl.), structural zero (str. zero).

| **A) IBD vs HC** | | | | **B) Week 0 vs week 40** | | | | |
| --- | --- | --- | --- | --- | --- | --- | --- | --- |
| **Taxa detected cut off 0.8W** | **W** | **CLR** | **Up in** | **Taxa detected cut off 0.8W** | **W** | **CLR** | **Up in** |  |
| *Actinomyces uncltr.* | str. zero | -0.602 | HC | *Bifidobacterium animalis* | str. zero | -0.488 | week 0 |  |
| *Rothia uncltr.* | str. zero | -0.623 | HC | *Barnesiella unclassified* | str. zero | -0.199 | week 0 |  |
| *Eggerthellaceae uncltr.* | str. zero | 0.052 | IBD | *Rikenellaceae RC9 gut group uncltr.* | str. zero | -0.536 | week 0 |  |
| *Bacteroides uncltr. Bacteroides* | str. zero | 0.101 | IBD | *Parabacteroides johnsonii* | str. zero | 0.108 | week 40 |  |
| *Bacteroides uncltr. bacterium* | 74 | 0.815 | IBD | *Parabacteroides uncltr.* | str. zero | 0.347 | week 40 |  |
| *Prevotellaceae NK3B31 group uncltr.* | str. zero | 0.191 | IBD | *Bilophila uncltr.* | str. zero | -0.024 | week 0 |  |
| *Rikenellaceae RC9 gut group uncltr.* | str. zero | -0.160 | HC | *Faecalitalea uncltr.* | str. zero | 0.327 | week 40 |  |
| *Parabacteroides johnsonii* | str. zero | 0.032 | IBD | *Blautia unclassified* | str. zero | 0.200 | week 40 |  |
| *Catenibacterium uncltr.* | str. zero | 0.040 | IBD | *Coprococcus uncltr.* | str. zero | 0.389 | week 40 |  |
| *Faecalitalea uncltr.* | str. zero | -0.123 | HC | *Dorea uncltr.* | str. zero | -0.361 | week 0 |  |
| *Enterococcus faecalis* | str. zero | -0.323 | HC | *Lachnoclostridium uncltr.* | str. zero | -0.407 | week 0 |  |
| *Enterococcus faecium* | str. zero | -0.070 | HC | *Roseburia uncltr.* | str. zero | -0.601 | week 0 |  |
| *Lactobacillus rhamnosus* | str. zero | -0.202 | HC | *Eubacterium hallii group uncltr.* | str. zero | -0.179 | week 0 |  |
| *Pediococcus unclassified* | str. zero | -0.240 | HC | *Ruminococcus gnavus group uncltr.* | str. zero | 0.719 | week 40 |  |
| *Streptococcus parasanguinis* | str. zero | -0.101 | HC | *Ruminococcus uncltr.* | str. zero | -0.214 | week 0 |  |
| *Christensenellaceae R-7 group uncltr.* | str. zero | -0.003 | HC | *Morganella morganii* | str. zero | -0.118 | week 0 |  |
| *Lachnoclostridium unclassified* | str. zero | 0.175 | IBD | *Akkermansia uncltr.* | str. zero | 0.494 | week 40 |  |
| *Lachnospiraceae NK4A136 group uncltr.* | str. zero | 0.090 | IBD | *Nakaseomyces unidentified* | str. zero | 0.289 | week 40 |  |
| *Roseburia unclassified* | str. zero | -0.048 | HC | *Diutina catenulata* | str. zero | 0.515 | week 40 |  |
| *Eubacterium ruminantium group uncltr.* | str. zero | 0.162 | IBD |  |  |  |  |  |
| *Ruminococcus gnavus group uncltr.* | str. zero | 0.515 | IBD |  |  |  |  |  |
| *Lachnospiraceae uncltr.* | str. zero | 0.165 | IBD |  |  |  |  |  |
| *Oscillospiraceae NK4A214 group uncltr.* | str. zero | 0.176 | IBD |  |  |  |  |  |
| *Oscillospiraceae UCG-002 uncltr.* | str. zero | -0.394 | HC |  |  |  |  |  |
| *Oscillospiraceae UCG-005 uncltr.* | str. zero | 0.114 | IBD |  |  |  |  |  |
| *Faecalibacterium uncltr.* | 72 | -0.893 | HC |  |  |  |  |  |
| *Subdoligranulum uncltr.* | 81 | -1.112 | HC |  |  |  |  |  |
| *Anaeroglobus uncltr.* | str. zero | -0.163 | HC |  |  |  |  |  |
| *Dialister unclassified* | str. zero | -0.284 | HC |  |  |  |  |  |
| *Dialister uncltr.* | str. zero | -0.180 | HC |  |  |  |  |  |
| *Megasphaera uncltr.* | str. zero | 0.003 | HC |  |  |  |  |  |
| *Klebsiella unclassifed* | str. zero | -0.046 | HC |  |  |  |  |  |
| *Morganella morganii* | str. zero | -0.138 | HC |  |  |  |  |  |
| *Unassigned* | 20 | -1.304 | IBD |  |  |  |  |  |
| *Fungi unclassified* | str. zero | -0.158 | IBD |  |  |  |  |  |
| *Cladosporium unidentified* | str. zero | -0.291 | IBD |  |  |  |  |  |
| *Dothideales unclassified* | str. zero | -0.080 | IBD |  |  |  |  |  |
| *Pichia unclassified* | str. zero | -0.334 | IBD |  |  |  |  |  |
| *Pichia membranifaciens* | str. zero | -0.295 | IBD |  |  |  |  |  |
| *Nakaseomyces unidentified* | str. zero | -0.195 | IBD |  |  |  |  |  |
| *Saccharomyces cerevisiae* | 19 | 2.401 | HC |  |  |  |  |  |
| *Boletaceae unclassified* | str. zero | -0.103 | IBD |  |  |  |  |  |
| *Cystobasidiomycetes unclassified* | str. zero | -0.180 | IBD |  |  |  |  |  |
| *Sporobolomyces unclassified* | str. zero | -0.197 | IBD |  |  |  |  |  |
| *Trichosporonaceae unclassified* | str. zero | -0.193 | IBD |  |  |  |  |  |
| *Wallemia unidentified* | str. zero | -0.146 | IBD |  |  |  |  |  |
| *Fungi unidentified* | str. zero | 0.036 | HC |  |  |  |  |  |
