# Supplementary material for: Association between ustekinumab therapy and changes in specific anti-microbial response, serum biomarkers, and microbiota composition in patients with IBD: A pilot study
Source: PLoS One. 2022 Dec 30;17(12):e0277576. doi: 10.1371/journal.pone.0277576 (PMC9803183; doi:10.1371/journal.pone.0277576)
Supplement: S16 Table — None of the alpha diversity metrics show a significant difference in the skin microbiome of IBD patients between the baseline (week 0) and endpoint (week 40) of the ustekinumab therapy A). Same results were obtained when week was fitted as a continuous variable B). Values for test statistics, associated degrees of freedom (DF), and resulting p values and q values with correction for multiple hypothesis testing (false discovery rate) are shown. (DOCX) [file pone.0277576.s018.docx]

**Supplementary Table 16:** Temporal variation of microbial skin alpha diversity of patients with IBD treated with ustekinumab. None of the alpha diversity metrics show a significant difference in the skin microbiome of IBD patients between the baseline (week 0) and endpoint (week 40) of the ustekinumab therapy **A)**. Same results were obtained when week was fitted as a continuous variable **B)**. Values for test statistics, associated degrees of freedom (DF), and resulting *p* values and *q* values with correction for multiple hypothesis testing (false discovery rate) are shown.

|  | **A) Temporal variation (categorical)** | | | | **B) Temporal variation (continuous)** | | | |
| --- | --- | --- | --- | --- | --- | --- | --- | --- |
| **Distance** | **DF** | **Test statistics** | ***p* value** | ***q* value** | **DF** | **Test statistics** | ***p* value** | ***q* value** |
| Shannon entropy | 1 | 0.151 | 0.883 | 0.883 | 1 | 1.132 | 0.265 | 0.354 |
| Chao1 | 1 | 0.623 | 0.546 | 0.728 | 1 | 1.451 | 0.154 | 0.308 |
| ASVs | 1 | 0.909 | 0.383 | 0.728 | 1 | 1.693 | 0.098 | 0.308 |
| Faith's PD | 1 | -3.721 | 0.020 | 0.081 | 1 | 0.199 | 0.844 | 0.844 |
