# Supplementary material for: Association between ustekinumab therapy and changes in specific anti-microbial response, serum biomarkers, and microbiota composition in patients with IBD: A pilot study
Source: PLoS One. 2022 Dec 30;17(12):e0277576. doi: 10.1371/journal.pone.0277576 (PMC9803183; doi:10.1371/journal.pone.0277576)
Supplement: S17 Table — MDMR testing differences in community composition between week 0 and week 40 A) or using week as a continuous variable B). Values for test statistics, associated degrees of freedom (DF) and resulting p values and q values with correction for multiple hypothesis testing (false discovery rate) are shown. (DOCX) [file pone.0277576.s019.docx]

**Supplementary Table 17:** Results of multivariate distance matrix regression (MDMR) testing for variation in the skin microbiome composition of patients with IBD during ustekinumab treatment. MDMR testing differences in community composition between week 0 and week 40 A) or using week as a continuous variable B). Values for test statistics, associated degrees of freedom (DF) and resulting *p* values and *q* values with correction for multiple hypothesis testing (false discovery rate) are shown.

|  | **A) Temporal variation (categorical)** | | | | **B) Temporal variation (continuous)** | | | |
| --- | --- | --- | --- | --- | --- | --- | --- | --- |
| **Distance** | **DF** | **Test statistic** | ***p* value** | ***q* value** | **DF** | **Test statistic** | ***p* value** | ***q* value** |
| Bray-Curtis | 1 | 1.0327 | 0.3829 | 0.5105 | 1 | 1.8884 | 0.0467 | 0.0935 |
| Jaccard | 1 | 7.2053 | < 0.0001 | < 0.0001 | 1 | 1.4653 | 0.0451 | 0.0935 |
| unweighted UniFrac | 1 | 2.8104 | 0.0060 | 0.0119 | 1 | 1.3067 | 0.1350 | 0.1800 |
| weighted UniFrac | 1 | 0.6706 | 0.5480 | 0.5480 | 1 | 1.3839 | 0.2036 | 0.2036 |
