# Supplementary material for: Association between ustekinumab therapy and changes in specific anti-microbial response, serum biomarkers, and microbiota composition in patients with IBD: A pilot study
Source: PLoS One. 2022 Dec 30;17(12):e0277576. doi: 10.1371/journal.pone.0277576 (PMC9803183; doi:10.1371/journal.pone.0277576)
Supplement: S18 Table — The table shows differentially abundant taxa determined by ANCOM2.1 between the baseline (week 0) and the endpoint (week 40) of the study on the skin of patients with IBD treated with ustekinumab. W-statistics and centred log ratios (CLR) are shown. Wmax was 23 and a 0.8W cut-off was chosen. Structural zero (str. zero). (DOCX) [file pone.0277576.s020.docx]

**Supplementary Table 18:** Taxa found on the skin of IBD patients that are differentially abundant between the baseline and endpoint of the study. The table shows differentially abundant taxa determined by ANCOM2.1 between the baseline (week 0) and the endpoint (week 40) of the study on the skin of patients with IBD treated with ustekinumab. W-statistics and centred log ratios (CLR) are shown. Wmax was 23 and a 0.8W cut-off was chosen. Structural zero (str. zero).

| **Taxa detected cut off 0.8W** | **W** | **CLR** | **Up in** |
| --- | --- | --- | --- |
| *Cutibacterium unclassified* | str. zero | 1.249 | week 40 |
| *Prevotella uncultured* | str. zero | -1.977 | week 0 |
| *Streptococcus salivarius* | str. zero | 0.553 | week 40 |
| *Acinetobacter unclassified* | str. zero | 0.651 | week 40 |
